# RASopathies: unraveling mechanisms with animal models

**DOI:** 10.1242/dmm.020339

**Published:** 2015-08-01

**Authors:** Granton A. Jindal, Yogesh Goyal, Rebecca D. Burdine, Katherine A. Rauen, Stanislav Y. Shvartsman

**Affiliations:** 1 Department of Chemical and Biological Engineering, Princeton University, Princeton, NJ 08544, USA; 2 Lewis-Sigler Institute for Integrative Genomics, Princeton University, Princeton, NJ 08544, USA; 3 Department of Molecular Biology, Princeton University, Princeton, NJ 08544, USA; 4 Department of Pediatrics, MIND Institute, Division of Genomic Medicine, University of California, Davis, Sacramento, CA 95817, USA

**Keywords:** Ras-MAPK, Developmental disorders, *Drosophila*, Zebrafish, Mice, Drug target

## Abstract

RASopathies are developmental disorders caused by germline mutations in the Ras-MAPK pathway, and are characterized by a broad spectrum of functional and morphological abnormalities. The high incidence of these disorders (∼1/1000 births) motivates the development of systematic approaches for their efficient diagnosis and potential treatment. Recent advances in genome sequencing have greatly facilitated the genotyping and discovery of mutations in affected individuals, but establishing the causal relationships between molecules and disease phenotypes is non-trivial and presents both technical and conceptual challenges. Here, we discuss how these challenges could be addressed using genetically modified model organisms that have been instrumental in delineating the Ras-MAPK pathway and its roles during development. Focusing on studies in mice, zebrafish and *Drosophila*, we provide an up-to-date review of animal models of RASopathies at the molecular and functional level. We also discuss how increasingly sophisticated techniques of genetic engineering can be used to rigorously connect changes in specific components of the Ras-MAPK pathway with observed functional and morphological phenotypes. Establishing these connections is essential for advancing our understanding of RASopathies and for devising rational strategies for their management and treatment.

## Abstract (precis)

**Summary:** Developmental disorders caused by germline mutations in the Ras-MAPK pathway are called RASopathies. Studies with animal models, including mice, zebrafish and *Drosophila*, continue to enhance our understanding of these diseases.

## Introduction

**Fig. 1.**
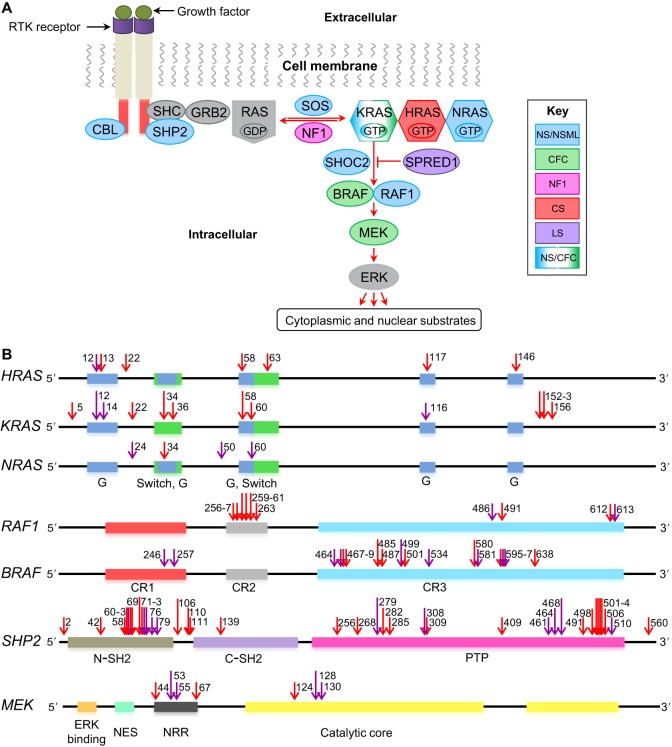
**The Ras-MAPK signaling pathway and associated mutations.** (A) Schematic of the Ras-MAPK signaling pathway. Proteins commonly mutated in RASopathies, color coded to represent different syndromes: Noonan syndrome (NS; blue), cardio-facio-cutaneous syndrome (CFC; green), neurofibromatosis type 1 (NF1; magenta), Costello syndrome (CS; red), Legius syndrome (LS; purple). (B) Positions of mutations in certain genes that encode components of the Ras-MAPK pathway. Purple arrows indicate where a mutation has been modeled in animals; red arrows indicate where it has not. Colored boxes represent regions in the genes that encode key protein domains. In the RAS proteins, the G regions (blue) form the nucleotide-binding site, and the switch regions (green) change conformation between the inactive and active states. In the RAF proteins, the CR1 region (red) contains a Ras-binding domain, the CR2 (gray) and CR3 (turquoise) regions associate with 14-3-3 proteins (a family of key regulatory proteins expressed in all eukaryotic cells). The CR2 region is also a site of regulatory phosphorylation. In the SHP2 protein, structural features include the N (brown) and C (purple) terminal Src homology 2 (SH2) domains, and a protein tyrosine phosphate (PTP) domain (pink). In the MEK protein, key protein domains include the negative regulatory region (NRR; black), the MAPK-binding site (ERK binding; orange), the nuclear export signal (NES; green) and the catalytic core (yellow). Numbers near the arrows indicate the protein residues that are mutated (see supplementary material Table S1 for more details). RTK, receptor tyrosine kinase.

The Ras pathway is a highly conserved cascade of protein-protein interactions and phosphorylation that controls a wide range of processes in adult and developing tissues (Fig. 1A) (Kolch, 2000). This cascade is triggered by signals from cell-surface receptors and culminates in the activation of the mitogen-activated protein kinase (MAPK), an enzyme with hundreds of substrates, including transcription factors and regulators of cell shape, apoptosis and metabolism (Boulton et al., 1990; Futran et al., 2013). The Ras pathway is active from the earliest stages of embryogenesis and is essential for tissue homeostasis in the adult (Futran et al., 2013; Jiang et al., 2011). Given its ubiquitous role in tissue regulation, it is not surprising that mutations in essentially every component of this pathway can lead to disease. Strong gain-of-function (GOF) mutations that arise in somatic tissues can result in multiple types of cancer (Hanahan and Weinberg, 2011). As a consequence, the components of the Ras pathway are vitally important drug targets in oncology.

More recently, new mutations that hyperactivate the Ras pathway have been discovered in the germline and linked to human developmental disorders that are collectively known as RASopathies (Rauen, 2013). Individuals with these conditions, which are estimated to affect about 1/1000 human births (Rauen, 2013), are characterized by pleiotropic phenotypes, including heart defects, short stature, neurocognitive impairment, craniofacial malformations and a predisposition to developing cancer. Since the identification of the first RASopathy, neurofibromatosis type 1 (NF1), which is caused by inactivation of neurofibromin 1, several other syndromes have been associated with mutations in the core components in the Ras-MAPK pathway (Fig. 1A). These include Noonan syndrome (NS), Noonan syndrome with multiple lentigines (NSML), formerly called LEOPARD syndrome, Costello syndrome (CS), cardio-facio-cutaneous syndrome (CFC), Legius syndrome (LS) and capillary malformation–arteriovenous malformation syndrome (CM-AVM) (Rauen, 2013). To date, hundreds of mutations have been described by molecular and genetic studies of these developmental abnormalities (Aoki et al., 2008) (Fig. 1B and supplementary material Table S1), and more continue to be documented in patient-specific sequencing projects (Chen et al., 2014a).

**Fig. 2.**
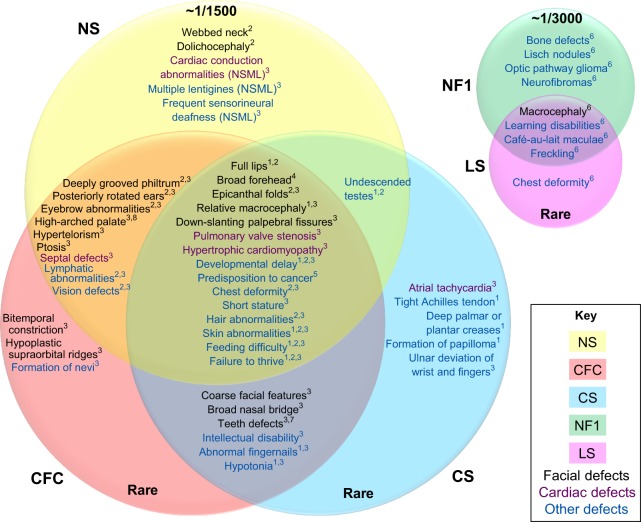
**Venn diagrams for disease phenotypes of the RASopathies.** Venn diagrams for common phenotypes of RASopathies are clustered accordingly. Colored circles represent different syndromes [Noonan syndrome (NS), yellow; cardio-facio-cutaneous syndrome (CFC), red; Costello syndrome (CS), blue; neurofibromatosis type 1 (NF1), green; Legius syndrome (LS), pink]; colored text indicates defect type (facial phenotypes are shown in black, cardiac defects are shown in purple, and developmental and other defects are in blue). The symptoms listed where circles overlap are common to different syndromes. The numbers indicate the reference number: ^1^Gripp and Lin, 2012, ^2^Roberts et al., 2013, ^3^Pierpont et al., 2014, ^4^Rauen, 2013, ^5^Kratz et al., 2011, ^6^Brems and Legius, 2013, ^7^Goodwin et al., 2014, ^8^Allanson, 1987. The prevalence of the syndrome in the population is indicated as a ratio, or by the “Rare” classification, meaning less than 1000 cases worldwide.

Although each RASopathy has a set of unique characteristics, individuals with distinct and different mutations in their Ras-MAPK pathway components do share some substantial morphological and behavioral phenotypes with each other (Fig. 2). Systematic efforts are underway to characterize the many postnatal abnormalities that are associated with human RASopathies, which will be essential for their proper diagnosis and for the development of rational strategies for their management and treatment. Specifically, a number of recent publications discuss the possibility of treating human RASopathies with drugs that were originally designed to be anticancer therapeutics, such as inhibitors of MAPK activation (Anastasaki et al., 2012; Chen et al., 2010; Li et al., 2005; Wang et al., 2012). In parallel with these studies in humans, basic research into the RASopathies seeks to elucidate the mechanisms that contribute to the emergence of their structural and functional phenotypes.

To explain the syndrome progression mechanistically, two interdependent lines of research have emerged. First, it is important to understand the changes that occur at the protein level. This question can be readily addressed by studies that use purified pathway components, both for wild-type and mutants, and cultured animal and mammalian cells (De Rocca Serra-Nédélec et al., 2012). For instance, it was shown that mutations in the SHP2 [Src homology 2 (SH2)-containing protein-tyrosine phosphatase] protein, which is commonly mutated in NS, relieve the intramolecular inhibition within this enzyme and lead to higher levels of pathway activation (Keilhack et al., 2005). At the same time, animal models can be used to investigate the effects of such mutations on tissue development and morphogenesis. Model organisms, such as *Mus musculus* (mice), *Drosophila melanogaster* (flies) and *Danio rerio* (zebrafish), have played a key role in delineating the core Ras-MAPK pathway and are now at the forefront of mechanistic studies into developmental abnormalities, including the RASopathies.

**Fig. 3.**
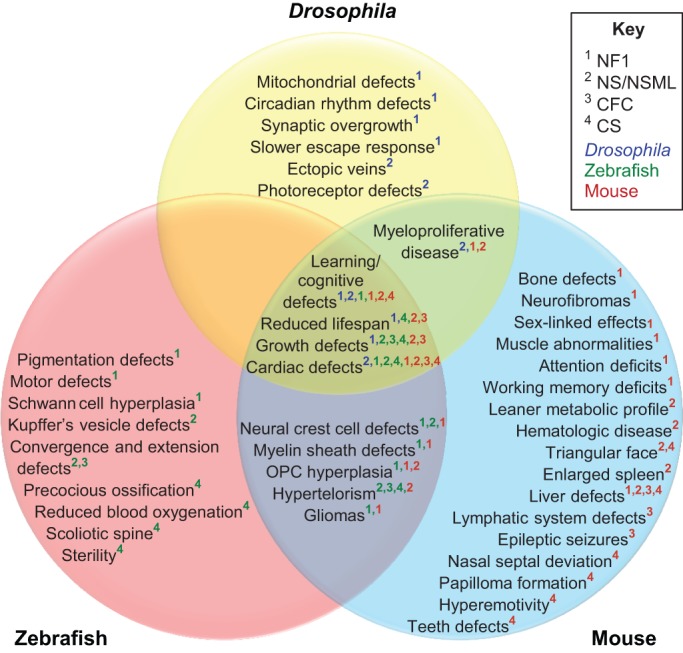
**A Venn diagram of phenotypes in animal models of RASopathies.** A Venn diagram of RASopathy-associated disease phenotypes that can be studied in animal models, showing phenotypes corresponding to those in *Drosophila* (yellow), zebrafish (red) and mouse (blue). Numbers indicate the modeled disease and the color code shows the organism used. The phenotypes listed where circles overlap are common to different models and/or syndromes. Cardio-facio-cutaneous syndrome (CFC); Costello syndrome (CS); Neurofibromatosis type 1 (NF1); Noonan syndrome (NS); Noonan syndrome with multiple lentigines (NSML); oligodendrocyte progenitor cell (OPC). The specific animal models are referenced in supplementary material Table S6.

Most of the characterized RASopathy mutations occur in conserved positions within pathway components, which implies that model organisms can be used to study the effects of specific mutations on developmental progress. Indeed, as discussed in this Review, many of the morphological and behavioral phenotypes of human RASopathies can be successfully phenocopied in model organisms (Fig. 3). Each of the model organisms presents unique advantages for the study of RASopathies. The mouse model is a commonly used mammalian model and can recapitulate many of the phenotypes observed in humans (supplementary material Tables S2, S3). The zebrafish model, with its translucent embryos, enables monitoring of the progression of developmental defects (supplementary material Table S4). Finally, *Drosophila*, an invertebrate model organism with a simple anatomy and a short life-cycle, supports high-throughput and quantitative studies that are more challenging to perform in vertebrate models (supplementary material Table S5). Here, we discuss what has been learned from animal models of RASopathies and pose open questions for future research aimed at further advancing our understanding of these conditions and at the design of approaches for their potential treatment.

## Neurofibromatosis type 1

**Fig. 4.**
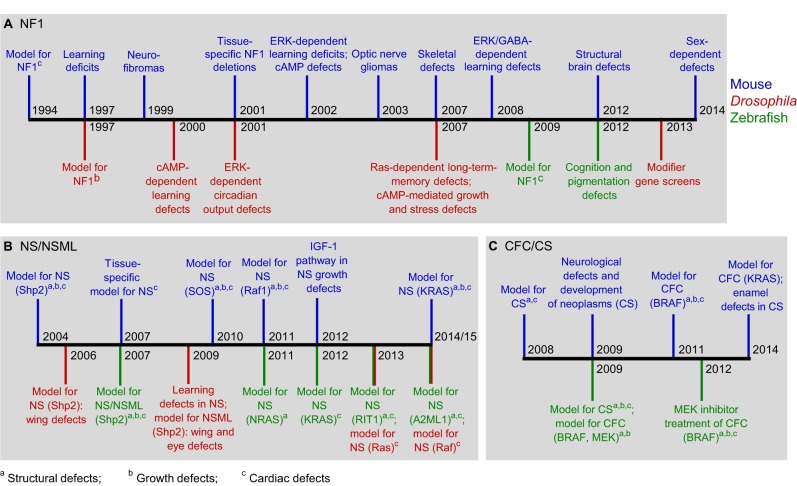
**Timeline of major developments in animal models for the RASopathies.** (A) Neurofibromatosis type 1 (NF1): 1994 (Brannan et al., 1994), 1997 (Silva et al., 1997; The et al., 1997), 1999 (Cichowski et al., 1999), 2000 (Guo et al., 2000), 2001 (Williams et al., 2001; Zhu et al., 2001), 2002 (Costa et al., 2002; Tong et al., 2002), 2003 (Bajenaru et al., 2003), 2007 (Ho et al., 2007; Kolanczyk et al., 2007), 2008 (Cui et al., 2008), 2009 (Padmanabhan et al., 2009), 2012 (Shin et al., 2012; Wang et al., 2012), 2013 (Walker et al., 2013), 2014 (Diggs-Andrews et al., 2014). (B) Noonan syndrome (NS) and Noonan syndrome with multiple lentigines (NSML): 2004 (Araki et al., 2004), 2006 (Oishi et al., 2006), 2007 (Jopling et al., 2007; Nakamura et al., 2007), 2009 (Oishi et al., 2009; Pagani et al., 2009), 2010 (Chen et al., 2010), 2011 (Wu et al., 2011), 2012 (De Rocca Serra-Nédélec et al., 2012; Razzaque et al., 2012), 2013 (Aoki et al., 2013; Yu et al., 2013b), 2014/15 (Hernández-Porras et al., 2014; Vissers et al., 2015; Yu et al., 2015). (C) Cardio-facio-cutaneous syndrome (CFC) and Costello syndrome (CS): 2008 (Schuhmacher et al., 2008), 2009 (Anastasaki et al., 2009; Chen et al., 2009; Santoriello et al., 2009; Viosca et al., 2009), 2011 (Urosevic et al., 2011), 2012 (Anastasaki et al., 2012), 2014 (Dalin et al., 2014; Goodwin et al., 2014). RIT1, Ras-like without CAAX 1; A2ML1, alpha-2-macroglobulin-like-1.

NF1 is caused by mutations in the *NF1* gene, which encodes neurofibromin 1, a Ras GTPase-activating protein (GAP), that acts as a negative regulator of Ras-MAPK signaling (Cawthon et al., 1990; Martin et al., 1990). NF1 affects about 1/3000 individuals worldwide (Williams et al., 2009). The most common features of NF1 include: the presence of benign tumors, such as neurofibromas and optic pathway gliomas (OPGs); bone malformations; mild neurocognitive impairments; cardiac defects; and a predisposition to cancer (Upadhyaya and Cooper, 2012; Williams et al., 2009). NF1 is the first and most studied RASopathy in animal models, and several of the key NF1-associated phenotypes have been recapitulated using model organisms (Fig. 4A). As a result of these studies, the molecular and cellular bases of several NF1-associated disease phenotypes are being progressively understood. Another related, although clinically distinct, rare disease is neurofibromatosis type 2 (NF2), which mainly causes tumor growth (Gutmann, 2001). However, it is caused by mutations in neurofibromin 2, which seems to link the actin cytoskeleton to cell-membrane-associated proteins (McClatchey and Giovannini, 2005). It is not a part of the canonical Ras-MAPK pathway and will not be covered in this Review.

The two initial mouse models for NF1 (*Nf1^+/Fcr^* and *Nf1^+/n31^*) were established more than 20 years ago, by inserting a neomycin cassette into the exonal region of *Nf1* (Brannan et al., 1994; Jacks et al., 1994). Whereas mouse lines that are heterozygous for these insertions show no phenotype, both homozygous lines display heart defects and neural crest defects, resulting in embryonic lethality at approximately 12.5-14 days of gestation (Brannan et al., 1994; Jacks et al., 1994; Lakkis et al., 1999). *Drosophila* models of NF1 have also existed for around 20 years, and were created using a P-transposon element insertion into the *Nf1* gene region (The et al., 1997). Unlike *Nf1^−/−^* mice, *Nf1^−/−^* flies are viable, but display post-embryonic general growth defects, such as reduced size of larvae and adults. Such general growth defects are also reported in individuals with NF1. *Drosophila* models of NF1 also display some unique phenotypes, including a reduced tendency to fly away when released (The et al., 1997; Tong et al., 2007). The more recently generated *nf1-*knockout models in zebrafish display pigmentation defects (Shin et al., 2012), whereas morpholino-induced (Box 1) zebrafish models display cardiac defects (Padmanabhan et al., 2009).

**Box 1. Genetic engineering techniques used to generate animal models of disease**

**Clustered regulatory interspaced short palindromic repeat (CRISPR)/Cas (CRISPR-associated) systems^1,2,3^:** in this system, the Cas9 nuclease cleaves genomic DNA at a specific locus guided by nucleotide complementarity to induce mutations. This system can be used to knock-in mutations into the endogenous locus of model organisms, including *Drosophila* and zebrafish. It holds promise to create more accurate models than those based on overexpression of the gene of interest.

**Cre-lox^1,2^:** Cre recombinase induces recombination of a DNA sequence flanked by *loxP* sites. This technique is widely used in mice to induce a variety of genetic alterations, including gene knockout and knock-in at the endogenous locus, with tissue-specific and temporal control.

**Gal4-UAS^3^:** transgenic lines of model organisms are generated to express the yeast transcription activator protein GAL4, which activates transcription once bound to the upstream activator sequence (UAS). In flies, this system has been used extensively to overexpress mutant protein variants.

**Heat-shock-based gene expression^1,2,3^:** this technique relies on the use of a heat-sensitive promoter to drive gene expression temporally. It has been used to control gene overexpression spatially and temporally, and can be used in combination with other techniques, such as Gal4-UAS and transposon-based methods.

**Morpholinos^2^:** oligomers of nucleotide analogs used to inhibit gene expression. In zebrafish, they are introduced via a microinjection needle and are used to knockdown NF1 protein expression.

**mRNA injection^2^:** transient overexpression of a protein of interest by introducing mRNA via a microinjection needle. In zebrafish, this system has been used to overexpress mutant protein variants early in development.

**TALENs^1,2,3^:** transcription activator-like effector nucleases (TALENs) are restriction enzymes generated by fusing a DNA-cleavage domain and a domain of a collection of nucleotide-binding motifs derived from TALE proteins. This system holds promise to knock-in mutations into the endogenous locus of model organisms, which creates more accurate models than those based on overexpression of the gene of interest.

**Transposon-based method^1,2,3^:** the use of a transposase to stably insert DNA flanked by specific sites into the genome. In model organisms, this approach has been used to overexpress mutant protein variants.

**ZFNs^1,2,3^:** zinc-finger nucleases (ZFNs) are restriction enzymes generated by fusing a DNA-cleavage domain and a zinc-finger domain that recognize a specific sequence in the genome. This system has been used to create a zebrafish model of NF1 and can also be used to introduce mutations into the endogenous locus of model organisms.

^1^Mouse; ^2^zebrafish; ^3^*Drosophila*.

### Heart defects

Because heart defects are primarily responsible for embryonic lethality in *Nf1^−/−^* mice, multiple studies have focused on further understanding this particular defect. *Nf1^−/−^* mice display Ras-MAPK-dependent defects in endocardial cushions, including excess proliferation and abnormal epithelial-mesenchymal transformation, resulting in obstructed blood flow (Ismat et al., 2006; Lakkis and Epstein, 1998). To bypass the problem of embryonic lethality, future modeling approaches could create biallelic *Nf1* deletions specifically in either endothelial or myocardial cells, the two major heart cell types. Embryos with endothelial-cell-specific biallelic deletion have defects in endocardial cushions and myocardium (Gitler et al., 2003), whereas embryos with myocardial-cell-specific biallelic deletion have a normal heart but display some adult-onset heart defects (Xu et al., 2009). The observation that endothelial cells contribute more to heart defects is consistent with findings in NS mouse models, as discussed later. Recent studies have also characterized heart defects, albeit less severe, in embryos with tissue-specific and ubiquitous monoallelic deletion of *Nf1* (Lasater et al., 2008, 2010; Xu et al., 2007).

### Cognitive deficits

Cognitive and learning deficits induced by loss of NF1 protein have been studied extensively in mice (Silva et al., 1997), *Drosophila* (Guo et al., 2000) and, more recently, zebrafish (Wolman et al., 2014), both at the behavioral and mechanistic level. The NF1 mouse models, in general, display a wide range of learning and cognitive deficits, including deficits in spatial learning (Silva et al., 1997), attention (Brown et al., 2010), working memory (Shilyansky et al., 2010), motor learning (van der Vaart et al., 2011), motivation (Wozniak et al., 2013) and social learning (Molosh et al., 2014). An initial study in *Nf1^+/−^* mice identified two phenotypes that resemble the symptoms observed in individuals with NF1 (Silva et al., 1997). First, learning deficits in *Nf1^+/−^* mice, as assessed in spatial memory tasks, can be compensated for by extended training. Second, despite widespread NF1 expression in the brain in wild-type mice, the heterozygous mutant mice showed no deficits in certain brain functions, such as associative learning, as assessed in the fear-conditioning paradigm in the animals.

Later studies focused on identifying the molecular basis of these deficits using pharmacological and genetic perturbations, including selective exonal deletions of *Nf1* (Costa et al., 2001). Several lines of evidence, including evidence for the overactivation of MAPK in the hippocampus of the mutant mice, implicate Ras-MAPK signaling in the increased phosphorylation of synapsin-1, a regulator of neurotransmitter release. This, in turn, leads to learning deficits caused by increased γ-aminobutyric acid (GABA) release (Costa et al., 2002; Cui et al., 2008; Guilding et al., 2007). Furthermore, tissue-specific deletions of *Nf1* revealed the specific cell types, i.e. inhibitory neurons, that are involved in spatial learning deficits (Cui et al., 2008). Both spatial learning deficits and attention deficits in the *Nf1*-mutant mice were rescued by attenuating Ras-MAPK signaling using pharmacological treatments, such as lovastatin, an inhibitor of p21Ras isoprenylation and activity, which has now been used in Phase 1 clinical trials on NF1-affected children (Acosta et al., 2011; Li et al., 2005). Importantly, treating NF1 mouse models with drugs that do not affect Ras-MAPK signaling, such as dopamine, also rescues their attention deficits (Brown et al., 2010, 2011; Diggs-Andrews et al., 2013). Given the heterogeneous nature of the cognitive and learning deficits of NF1 individuals, these findings present opportunities for exploring patient-specific therapeutic targets beyond Ras-MAPK-specific inhibitors.

*Drosophila* has also been used as a model for behavioral studies of NF1. *Nf1^−/−^* flies display neurocognitive deficits in olfactory learning and long-term memory, a result of abnormal cyclic adenosine monophosphate (cAMP) and Ras-MAPK signaling, respectively (Guo et al., 2000; Ho et al., 2007). Rescue studies identified the mushroom body (MB) neurons as being the specific cell type responsible for learning deficits in *Drosophila* (Buchanan and Davis, 2010), although more recent studies showed that cells outside of the MB neurons also contribute to NF1 memory defects (Gouzi et al., 2011; Walker et al., 2013). Furthermore, NF1 affects only the acquisition, but not the stability, of memories (Buchanan and Davis, 2010). In addition to learning deficits, *Nf1^−/−^* flies display abnormal circadian output in a Ras-MAPK-dependent manner (Williams et al., 2001). This suggests the possibility of sleep disturbances in individuals with RASopathies, an effect that could be explored by studies in the clinic.

### Structural and growth defects

Structural and growth defects, particularly skeletal phenotypes observed in individuals with NF1, have been studied extensively in mouse models. *Nf1^+/−^* mice display only mild bone defects, and are not a good model of NF1-associated skeletal malformations, which can be quite variable in individuals with NF1 (Yu et al., 2005). However, because *Nf1^−/−^* mice are embryonic lethal, follow-up studies focused on creating many tissue-specific biallelic deletions of *Nf1*. These models recapitulate several phenotypes observed in humans, including bowing of the tibia, impaired bone strength and short stature (de la Croix Ndong et al., 2014, 2015; El Khassawna et al., 2012; Kolanczyk et al., 2007; Kossler et al., 2011; Rhodes et al., 2013; Schindeler et al., 2008; Wang et al., 2011; Zhang et al., 2011). Some of these phenotypes might be caused by increased levels of pyrophosphate, a strong inhibitor of bone mineralization. This is due to increased Ras-MAPK signaling causing the enhanced expression of downstream genes that regulate pyrophosphate synthesis and extracellular transport (de la Croix Ndong et al., 2014).

In addition to skeletal phenotypes, mice with NF1-deficient brains display brain malformations, such as an enlarged corpus callosum, which has only recently been associated with severe learning deficits in a subset of individuals with NF1 (Wang et al., 2012). Importantly, studies in mice suggest that these defects are established as early as neonatal development, and that Ras-MAPK pathway inhibition during this developmental time window not only rescues the structural brain defects, but also improves the health of these mutant mice (Wang et al., 2012).

Two other features of NF1, general growth defects and reduced lifespan, have been observed in animal models of this disease. Studies in *Drosophila*, and more recently in mice, have also reported a role for NF1 protein in cAMP-regulated and Ras-MAPK-regulated mitochondrial function (Sullivan et al., 2014; Tong et al., 2007), although the precise molecular basis for this role remains unknown. For instance, despite compelling evidence that cAMP has a role in the growth defects observed in *Nf1*^–/–^ flies, it is still unclear whether it acts directly or indirectly (Hannan et al., 2006; Tong et al., 2002; Walker et al., 2006). These studies lay the foundation for further investigations into the signaling basis of the metabolic consequences of *NF1* deletions in humans.

### Benign tumors

About 15% of children with NF1 have poor vision because of benign OPGs (Listernick et al., 1997). Early studies of this effect in the *Nf1^+/−^* mouse model reported increased astrocyte proliferation but not OPG formation (Bajenaru et al., 2001; Bennett et al., 2003; Gutmann et al., 1999; Rizvi et al., 1999). However, it was later determined that OPGs can be induced by combining a monoallelic *Nf1* deletion with a biallelic deletion of *Nf1* in astrocytes (Bajenaru et al., 2002, 2003; Solga et al., 2014; Zhu et al., 2001, 2005). Furthermore, this effect was traced to specific cells of origin: neural stem cells in the third ventricle (Lee et al., 2012). Research is also underway to identify the specific cells that aid the neural stem cells in OPG formation (Daginakatte and Gutmann, 2007; Daginakatte et al., 2008). The precise biochemical bases of these cell- and tissue-level developmental defects are not fully understood, but several lines of evidence suggest that OPG formation is associated with increased phosphatidylinositol 3-kinase (PI3K)-Akt-mammalian target of rapamycin (mTOR) signaling, and not with Ras-MAPK signaling (Banerjee et al., 2011a,b; Dasgupta et al., 2005; Hegedus et al., 2007). Finally, mouse models of malignant gliomas are induced by a monoallelic knockout of *Nf1* and of other tumor suppressor genes, including *p53* (for further reading, see Chen et al., 2012).

Neurofibromas, which are benign peripheral nerve sheath tumors, come in a variety of forms, including dermal neuroﬁbromas, which are associated with individual nerves, and larger plexiform neuroﬁbromas that arise from connected nerve bundles. These neurofibroma types were studied using tissue-specific, biallelic, deletions of *Nf1* in *Nf1^+/−^* mice (Chen et al., 2014b; Joseph et al., 2008; Le et al., 2009, 2011; Mayes et al., 2011; Ribeiro et al., 2013; Wu et al., 2008; Zhu et al., 2002). As with the OPGs, further research narrowed down the specific cell types that contribute to the effects induced by bi- and mono-allelic deletion of *Nf1* (Yang et al., 2008; for further reading, see Gutmann, 2014). Older individuals with NF1 have an increased risk of developing malignant peripheral nerve sheath tumors (MPNSTs). *Nf1^+/−^* mice only display MPNSTs when the monoallelic *Nf1* deletion is combined with the loss of another tumor suppressor gene, such as *p53*, indicating that loss of these tumor suppressor alleles is the rate-limiting step in neurofibroma formation (Cichowski et al., 1999; Reilly et al., 2006; Rosenbaum et al., 1997; Vogel et al., 1999). MEK (MAPK kinase) inhibitors and cucurbitacin-I, an inhibitor of signal transducer and activator of transcription-3 (STAT3) signaling, have shown promise in decreasing the size of neurofibromas and of MPNSTs (Banerjee et al., 2010; Jessen et al., 2013). It was also found that MEK and mTOR inhibitors work synergistically to reduce MPNST size and increase lifespan in mice (Watson et al., 2014a).

In summary, most of the key features observed in individuals with NF1 can be successfully phenocopied in mouse models. For skeletal phenotypes and OPG formation, mechanistic models that link NF1 loss to biochemical effects, cell proliferation and functional outcomes are beginning to be formulated. The *Drosophila* models have contributed towards identifying key regulatory roles of NF1 in cAMP signaling and mitochondrial metabolism. The zebrafish model has not been used as extensively, but a recent model recapitulates neurocognitive deficits (Wolman et al., 2014) and is likely to be pursued further.

## Noonan syndrome and Noonan syndrome with multiple lentigines

NS is caused primarily by GOF mutations in the protein tyrosine phosphatase, non-receptor type 11 (*PTPN11*) gene, which encodes SHP2. NS might also be caused by GOF mutations in other Ras-MAPK-pathway components, such as Kirsten rat sarcoma viral oncogene homolog (*KRAS*), neuroblastoma RAS viral (v-ras) oncogene homolog (*NRAS*), Raf-1 proto-oncogene (*RAF1*), son of sevenless homolog 1 (*SOS1*) and soc-2 suppressor of clear homolog (*SHOC2*), and loss-of-function (LOF) mutations in Cbl proto-oncogene (*CBL*) (Fig. 1). Individuals with NS are characterized by craniofacial malformations, congenital heart defects, myeloproliferative disease, growth and neurocognitive delay, and an increased risk of developing cancer (Roberts et al., 2013). Missense mutations in *SHP2* and, in some cases, *RAF1* have been associated with NSML, which is discussed later in this section. Individuals with NSML have most of the clinical features observed in individuals with NS. In addition, they display increased penetrance of hypertrophic cardiomyopathy and lentigines (tan or brown macules on the skin) (Sarkozy et al., 2008). Animal models, particularly mice, with NS mutations successfully model many behavioral and phenotypic aspects of individuals with NS (Fig. 3), and have enhanced our understanding of this disease's etiology at the level of signaling and metabolism.

The sequencing of individuals with NS continue to identify multiple mutations in the N-SH2 and PTP domains of SHP2, which are responsible for auto-inhibition and for the intrinsic activity of the enzyme (Yu et al., 2013a), respectively. The effects of these mutations were explored in studies with human cultured cells stimulated with exogenously added growth factors, which induce a transient pulse of Ras-MAPK activation. Overexpression of mutant SHP2 protein increased the level of MAPK activation in these assays (Yu et al., 2014). The magnitude of this effect depends on the position and nature of the mutation. For instance, one study suggests that SHP2-D61G and SHP2-N308D are among the strongest and weakest activating mutations of SHP2 catalytic activity, respectively (Keilhack et al., 2005). Animal models have been generated for a few of these mutations, and these models show several, often overlapping, NS phenotypes. In some cases, the differences in the enzymatic activities of SHP2 that are observed *in vitro* are recapitulated in mice carrying these mutations. For instance, two copies of the *Shp2-N308D* allele are needed to produce similar heart defects to those seen in mice carrying only one copy of the *Shp2-D61G* allele (Araki et al., 2009). In general, however, more work is required to test whether the ranking of mutations revealed by studies in cultured cells reflects their effects *in vivo*.

Mice and zebrafish models of NS display comparable craniofacial malformations. The first mouse model to carry an NS-causing *Shp2* mutation developed a ‘triangular’ facial appearance and a larger width-to-length ratio of the skull. Studies of craniofacial development in these animals revealed that increased levels of Ras-MAPK signaling are present in specific tissues, such as the developing face, consistent with the positive role of SHP2 in Ras-MAPK activation (Araki et al., 2004). In fact, a later study showed that neural-crest-cell-specific expression of an NS-causing *SHP2* mutation causes similar craniofacial malformations, indicating that these cells are directly responsible for these defects (Nakamura et al., 2009). The larger width-to-length skull ratio is also observed in multiple studies of zebrafish models generated by mRNA injection of zebrafish *shp2* or human *NRAS* variants that carry NS- and NSML-causing mutations (Jopling et al., 2007; Runtuwene et al., 2011; Stewart et al., 2010) (see Box 1). These studies in mice and fish also model another common NS phenotype, hypertelorism (widely spaced eyes). Various NS mice and zebrafish models also display general growth defects, such as reduced weight and short stature (Araki et al., 2004; Hernández-Porras et al., 2014; Jopling et al., 2007). Recent molecular studies suggest that short stature results from lower than normal levels of insulin-like growth factor I (IGF-1), which is believed to be a downstream effect of increased Ras-MAPK activity (De Rocca Serra-Nédélec et al., 2012).

Heart malformations are one of the most extensively studied features in animal models of NS, and the associated cell types responsible for heart defects have been narrowed down significantly. The *Shp2*-mutant mice have multiple heart defects, including ventricular septal defects (VSDs), atrial septal defects (ASDs) and double-outlet right ventricle (DORV), all of which are observed in individuals with NS. However, of the myocardial and endocardial cells in the heart, only endocardial cells have increased MAPK activation (Araki et al., 2004). Prompted by this observation, later studies showed that mice with endocardial-cell-specific NS-causing mutant SHP2 expression could recapitulate most of the heart defects observed when this gene is ubiquitously expressed (Araki et al., 2009; Krenz et al., 2008). Myocardial-specific fetal expression of *SHP2* mutations causes a smaller subset of heart defects (Nakamura et al., 2007), but their postnatal expression does not cause any defects (Araki et al., 2009; Dalin et al., 2014; Nakamura et al., 2007), suggesting that endocardial cells are primarily responsible for the NS-related heart defects. These heart defects are similar to those observed in NF1 mouse models (Gitler et al., 2003).

Zebrafish models generated by mRNA injection of zebrafish *shp2* or human *KRAS* variants that carry NS- and NSML-causing mutations develop heart defects, including enlarged atriums at 3 days post-fertilization (dpf) and excess fluid buildup at 5 dpf (Jopling et al., 2007; Miura et al., 2013; Razzaque et al., 2012). The translucent nature of zebrafish embryos allows a mechanistic understanding of the origins of these defects to be established by tracking organ development, starting as early as 1 dpf. A recent study reported that cardiac jogging, the asymmetric leftward migration of heart cells, becomes randomized in NS and NSML zebrafish models. This effect was attributed to defective cilia formation in Kupffer's vesicle, an organ responsible for directing left-right asymmetry in zebrafish (Bonetti et al., 2014).

Knock-in and virally transfected *Shp2*-mutant mouse models were also used to characterize behavioral aspects of NS, such as spatial learning and memory deficits. Although the cellular mechanisms of such deficits differ from NF1 mouse models, treatment with lovastatin, which has been extensively used in NF1 mouse models, reversed the NS-associated neurocognitive impairments (Costa-Mattioli, 2014; Lee et al., 2014). In parallel, *Drosophila* models [made using the Gal4-UAS system (Box 1)] for GOF mutations in *corkscrew* (*csw*), the fly ortholog of *PTPN11*, display long-term memory deficits (Pagani et al., 2009). Memory-inducing training generates a transient pulse of Csw-dependent Ras-MAPK signaling in fly heads. Biochemical analysis of fly heads after training revealed that memory deficits result from a prolonged interval of MAPK activity in *csw* GOF mutants, as compared to the activity in wild-type flies.

Mouse and *Drosophila* models of NS have been used to study other syndrome phenotypes, such as abnormal myelination (Ehrman et al., 2014), increased angiogenesis (Wang et al., 2009), reduced apoptosis (Gafuik and Steller, 2011) and myeloproliferative disease (Araki et al., 2004; Mohi et al., 2005). These studies have begun to provide a mechanistic understanding of disease development. For example, a study on the *Shp2*-mutant mice established that aberrantly accelerating hematopoietic stem cell cycling leads to myeloproliferative disease (Xu et al., 2010).

Efforts have also been made to generate mouse models of NS arising from the GOF mutations in other pathway components such as *KRAS*, *RAF1* and *SOS1*, a Ras guanine nucleotide exchange factor (GEF). Whereas the *Kras*-mutant mice largely phenocopy the *Shp2*-mutant mice (Hernández-Porras et al., 2014; Tuveson et al., 2004), the *Sos1*- and *Raf1*-mutant mice display some unique features. For instance, the *Sos1*-mutant mice develop unique cardiac phenotypes, such as the growth of excessive connective tissue and defects in different heart valves to those affected in the *Shp2*-mutant mice. These characteristic defects are thought to be due to the additional activation of Ras-related C3 botulinum toxin substrate (RAC1) and STAT3, compared to just Ras-MAPK signaling in the *Shp2*-mutant mice (Chen et al., 2010). Similarly, the *Raf1*-mutant mice and the *Ras*-mutant *Drosophila* display hypertrophic cardiomyopathy, typically not seen in the *Shp2* mutants (Wu et al., 2011, 2012; Yu et al., 2013b, 2015). From these studies, it is clear that many phenotypes are specific to certain proteins in the pathway.

Most animal studies of NS have focused on GOF mutations of *SHP2*, but LOF *SHP2* mutations in humans, which result in NSML, show some clear phenotypic differences to NS-causing GOF *SHP2* mutations. Mice with LOF mutations in *Shp2* consistently exhibit hypertrophic cardiomyopathy, a phenotype that is less penetrant in the GOF mutant mice. This is consistent with the observed phenotypes in individuals with these mutations (Edwards et al., 2015; Marin et al., 2011; Schramm et al., 2012). Furthermore, in contrast to the hyperactivation of Ras-MAPK signaling in mouse models of NS, Akt-mTOR signaling was implicated in NSML phenotypes. This observation implies that mTOR inhibitors could be used to treat individuals with NSML, whereas MEK inhibitors might be more appropriate for individuals with NS.

A recent mouse model study focused on the metabolic consequences of an NSML-associated mutation in *SHP2*, and found impaired adipogenesis and a better metabolic profile, which result in reduced body mass index (BMI) (Tajan et al., 2014). Prompted by these observations in mouse models, the same study examined BMI in a French cohort of individuals with NSML. Strikingly, they displayed impaired adipogenesis leading to reduced BMI, which underscores the potential of animal models to explore the molecular markers associated with this syndrome.

To summarize, findings from NS mouse models indicate that, although there is a significant overlap between the phenotypes caused by different mutations in genes implicated in NS, there are certain detectable differences as well. The process of associating these differences with the underlying signaling and metabolic pathways has begun, and further progress in this direction should lead to the development of new patient-specific drugs for NS. In addition, the zebrafish is a useful model for studying not only the final phenotypes but also their emergence during development. In parallel, *Drosophila* has mainly served as a system for the *in vivo* analysis of the biochemical effects of NS mutations.

## Cardio-facio-cutaneous syndrome

CFC syndrome is primarily caused by mutations in the B-Raf proto-oncogene (*BRAF*) and *MEK* genes, and, in some cases, the *KRAS* gene (Fig. 1). Although there is variability in the clinical phenotype among individuals with CFC syndrome, most have craniofacial malformations, congenital heart defects, musculoskeletal abnormalities and growth delay (Pierpont et al., 2014). Because CFC syndrome is a relatively rare disease, fewer animal models have been made and our mechanistic understanding of the origins of the observed phenotypes is still rudimentary. Existing mouse models of CFC syndrome recapitulate multiple human phenotypes and, together with the zebrafish models, show that MEK inhibitors, used to treat cancer, can ameliorate some of the effects of this disease.

Transgenic mouse models carrying various GOF mutations in *Braf*, all generated using Cre-lox technology (Box 1), show largely consistent phenotypes: the heterozygous *Braf*-mutant mice with the most prevalent CFC mutation, Q241R, display embryonic skeletal abnormalities, lymphatic defects, cardiac defects and liver necrosis (Inoue et al., 2014). Notably, these mutations cause embryonic lethality, but normal early development can be restored in mutant embryos by treating them prenatally with MEK inhibitors (Inoue et al., 2014), which is also the case for zebrafish models generated by mRNA injection of human *BRAF* variants with CFC-causing mutations (Anastasaki et al., 2009, 2012). Another mouse model of CFC syndrome that carries the L597V *Braf* mutation displays short stature, facial dysmorphia, cardiac enlargement and hypertrophic cardiomyopathy (Andreadi et al., 2012). These could be more accurate models to use for the molecular and phenotypic characterizations of CFC than the first mouse model of this disease, which was generated by expressing low levels of a constitutively active *Braf* allele (Urosevic et al., 2011).

Although we still need to understand the molecular origins of the defects in the existing CFC models, we also need to model other reported CFC-associated mutations, particularly in *MEK*, in order to further characterize the emergence and progression of CFC-related phenotypes. Zebrafish models were generated by mRNA injection of human *MEK* variants with CFC-causing mutations, but phenotypes relating to human-associated RASopathies have yet to be reported, although, as mentioned below, the zebrafish models develop an oval shape due to improper convergence and extension during gastrulation (Anastasaki et al., 2009). This defect has been used as a quantitative assay for the extent of overactive Ras-MAPK signaling (Bonetti et al., 2014; Runtuwene et al., 2011).

## Costello syndrome

CS is caused by mutations in the Harvey rat sarcoma viral oncogene homolog (*HRAS*) gene, with the majority of affected individuals carrying a mutation at the G12 position (Fig. 1). Not surprisingly, most animal models for CS have focused on this particular residue. Most CS individuals have characteristic craniofacial malformations, heart defects, growth and neurocognitive delay, musculoskeletal abnormalities, and cutaneous abnormalities, including papillomas – tumors that arise on the skin (Gripp and Lin, 2012). As with CFC, few animal models exist for this syndrome. Various mouse and zebrafish models of CS, however, do accurately phenocopy many of its human symptoms, including increased tumorigenesis in CS, compared to the other RASopathies.

Knock-in mouse models of CS were created using a constitutively active G12V variant, a rarely occurring mutation in CS, but a common cancer mutation. One set of mouse models included, in the 3′ untranslated region of *Hras*, an internal ribosome entry site followed by a β-Gal-neomycin resistance reporter, whereas the other set of mouse models effectively inserted the G12V mutation into the endogenous locus. Somewhat surprisingly, these models yielded a largely different set of phenotypes, except for craniofacial malformations and teeth defects, which occur in both (Chen et al., 2009; Schuhmacher et al., 2008). Both the homozygous and heterozygous mutant mice with the additional reporter are viable but display common CS phenotypes, such as facial dysmorphia, hypertrophic cardiomyopathy and hypertension (Schuhmacher et al., 2008). Furthermore, later studies characterized the presence of neurocognitive deficits, such as hyperemotivity and hypersensibility, although only in the homozygous mutant mice (Viosca et al., 2009). These mice, however, fail to recapitulate other CS symptoms, such as growth delay, skin defects and susceptibility to tumors. On the other hand, the transgenic mice without the additional reporter have high perinatal lethality and develop teeth defects, but not heart defects (Chen et al., 2009; Goodwin et al., 2014). Unlike the mice with the additional reporter, these mice develop tumors, including papillomas and angiosarcomas, a unique distinguishing feature of individuals with CS (Chen et al., 2009, 2014c). It is possible that the differences in phenotypes between the two models occur as a result of the interference of the additional reporter with the expression level and function of HRAS-G12V. Notably, activated MAPK levels in both mouse models are similar to the wild-type levels in all organs except the liver, where the level of activated MAPK was markedly increased (Chen et al., 2009; Schuhmacher et al., 2008). The origins of these tissue-specific effects are still unclear.

A zebrafish model for CS was generated using the Tol2 transposase to stably introduce the same *HRAS-G12V* variant into the zebrafish genome using both constitutive and heat-shock-inducible overexpression (Box 1). The adult zebrafish were characterized by their reduced size, scoliosis, reduced lifespan, smaller heart, craniofacial dysmorphology and tumorigenesis, all similar to human features of CS (Santoriello et al., 2009). However, the work reported a lack of activated MAPK or Akt signaling; thus, the molecular and cellular bases of these defects still largely remain unknown.

Taken together, many CS-associated developmental defects have been accurately recapitulated in mice and zebrafish, but not yet in *Drosophila*. Of note, these models express a constitutively active allele of *HRAS*, which is not the most common CS-causing allele of *HRAS*, which is just strongly activating (Sol-Church and Gripp, 2009). So far, biochemical studies of tissues in the mouse and zebrafish models of CS have not found consistently increased levels of either MAPK or Akt activation, and an explanation of this is currently lacking.

## Legius syndrome

LS is caused by complete LOF mutations in the Sprouty-related, EVH1 domain containing 1 (*SPRED1*) gene, which encodes a negative regulator of Ras-MAPK signaling (Brems et al., 2007). Individuals with LS have overlapping symptoms with NF1, although a few differentiating features were recently reported in some individuals (Brems and Legius, 2013; Stowe et al., 2012). So far, LS has been studied only in mice. However, these *Spred1*^−/−^ mice recapitulate several of the disease phenotypes, including shortened faces and deficits in learning and memory (Denayer et al., 2008; Inoue et al., 2005). They also display other phenotypes, including lower body weights, tail abnormalities and higher white blood cell counts, that are as yet not associated with LS (Denayer et al., 2008; Inoue et al., 2005; Phoenix and Temple, 2010). Although the potential effects of Ras-MAPK-pathway inhibitors have not been studied, this is a promising direction for future research.

## Discussion: future developments and open questions

More than 20 years since the first RASopathy animal model was established, much progress has been made in generating animal models that recapitulate many of the human phenotypes associated with these disorders. The progress made in characterizing the major phenotypic and molecular markers of the three main animal models of these disorders is summarized in Fig. 4. Most of the models generated to date have been mouse models of NF1. This is perhaps unsurprising, because NF1 models can be created by gene knockout, whereas other RASopathy models require the introduction of a specific mutation into the endogenous locus, which is technically more challenging to achieve. Animal models of NS and NSML have, however, become more prevalent in the past 10 years (Fig. 4B,C), compared with those generated for CS or CFC, potentially due to the higher prevalence of NS and NSML in the population resulting in increased research into these diseases. Other animal models, such as the nematode *Caenorhabditis elegans* and the frog *Xenopus laevis*, are also increasingly being used to model RASopathies, either to mimic a specific phenotype observed in humans (Langdon et al., 2012) or as an *in vivo* assay for investigating the activity of a mutant protein (Cordeddu et al., 2009; Flex et al., 2014).

With the exception of NF1, studies in zebrafish and *Drosophila* have so far relied on overexpression techniques such as mRNA injection and transposon-based methods (Box 1), either to mimic a specific phenotype observed in humans or to assay for the activity of the mutant protein *in vivo*. These studies have provided valuable information about the functions of mutant proteins. However, to accurately model a disease in heterozygous individuals it is more desirable to introduce mutations at the endogenous locus. This can be done using recently reported robust and efficient genome-editing techniques, including transcription activator-like effector nucleases (TALENs), zinc-finger nucleases (ZFNs) and the clustered regulatory interspaced short palindromic repeat (CRISPR)/CRISPR-associated system (Cas) (Box 1) (Gaj et al., 2013), which are optimized for different model organisms, such as *Drosophila* (Gratz et al., 2014) and zebrafish (Irion et al., 2014). Future studies will benefit from these techniques, which can ensure similar expression levels for both wild-type and mutant proteins, and can avoid potential dominant-negative effects caused by gene overexpression.

Certain mutant phenotypes that are unique to *Drosophila* and zebrafish do not correspond directly with those observed in humans, but they can nevertheless serve as useful quantitative assays for testing disease-associated mutations. For example, assays specific to *Drosophila*, such as Ras-MAPK-mediated wing and eye development, have provided a means to rank *in vivo* NS- and NSML-associated *SHP2* mutations, based on the severity of the phenotypes they produce (Oishi et al., 2006, 2009). Similarly, at 10 hpf, the oval shape of zebrafish embryos, which results from improper convergence and extension cell movements during gastrulation in mutant embryos (Jopling et al., 2007), has been used as a quantitative measure of activity of NS-causing *nras* mutations and of NS- and NSML-causing *shp2* mutations (Bonetti et al., 2014; Runtuwene et al., 2011).

We have reviewed a number of studies that provide a cellular basis for the structural and functional abnormalities caused by mutations associated with human RASopathies. These studies have revealed that RASopathy-associated mutations produce a broad range of functional effects, from the improper assignment of cell fates to changes in the patterns of cell migration and differentiation. How do these cell- and tissue-level changes emerge in the first place? Do they reflect significant changes in the spatiotemporal patterns of Ras-MAPK signaling? Answering these questions requires quantitative information about the wild-type signaling patterns that underlie numerous Ras-dependent developmental processes. Surprisingly, this important information is currently lacking, despite hundreds of studies on the developmental functions of the Ras-MAPK pathway. As a first step towards obtaining this information, we could enhance our current knowledge of MAPK signaling patterns during development by quantifying the duration, strength and variability of endogenous signaling patterns, thus making them more quantitative (Corson et al., 2003; Gabay et al., 1997; Krens et al., 2008).

Functional studies of RASopathy-associated mutations can also now rely on approaches to reconstruct developmental dynamics from ‘snapshots’ of MAPK phosphorylation in embryonic tissues fixed at different developmental stages (Lim et al., 2013), and on recently developed live reporters of MAPK activity (Fujioka et al., 2006; Regot et al., 2014). If successful, these approaches could be combined with genetic and genome-editing techniques to quantify the signaling changes induced by specific RASopathy mutations. A better biochemical characterization of the Ras-MAPK pathway dynamics in RASopathies could guide the development of pharmacological strategies to specifically modulate this pathway for therapeutic purposes.

With a few notable exceptions, human and animal model studies tend to focus on the end stages of developmental abnormalities. However, certain animal models offer opportunities to observe the progression of organ malformation; the zebrafish, for example, has a program of vertebrate development comparable to that in humans and transparent embryos that develop externally. Another setting where morphogenesis can be monitored closely is in organoids grown from induced pluripotent stem (IPS) cells. Because IPS cells can be derived from individuals with RASopathy (Carvajal-Vergara et al., 2010), it is possible to study developmental processes in organoids grown from the cells of an affected individual with a specific genotype. The usefulness of organoids for studying developmental processes has already been well demonstrated by the creation of organoids to study gut (Watson et al., 2014b) and brain (Lancaster et al., 2013) morphogenesis, using cells from individuals with affected organs.

Understanding how abnormalities caused by specific RASopathy-associated mutations develop during embryogenesis and postnatally can inform new approaches for the postnatal treatment of the RASopathies. As an example, a recent study established that reduced cerebellar size in an NF1 mouse model reflects postnatal functions of the Ras-MAPK pathway and its negative regulation by NF1. By pinpointing the time window during which this pathway controls cellular processes and the final size of the cerebellum, Sanchez-Ortiz et al. proposed that postnatal administration of the Ras-MAPK pathway inhibitor can normalize both MAPK signaling levels and cerebellar size (Sanchez-Ortiz et al., 2014). A similar approach, based on neonatal pharmacological treatment of NF1 mice using the MEK inhibitor PD0325901, has been used to rescue structural defects in the brain (Wang et al., 2012). As described above in the text, several studies have demonstrated that Ras-MAPK inhibitors, such a lovastatin, can reverse neurocognitive defects in an NF1 mouse model (Lee et al., 2014; Li et al., 2005), indicating a potential opportunity to test the therapeutic potential of these inhibitors further in small-scale trials with human NF1 patients (Acosta et al., 2011). Note, however, that because RASopathies are chronic conditions, their pharmacological treatment might require different strategies, which can be explored in a range of animal models.

In conclusion, studies in a range of experimental systems have demonstrated that many of the structural and functional abnormalities associated with human RASopathies can be successfully mimicked in model organisms. However, we are still far from a mechanistic picture that provides a clear link between any given mutation and the emerging defects. To establish this, we need to devise new and more sensitive assays for quantifying the effects of RASopathy mutations on Ras-MAPK signaling levels *in vivo*, to connect these changes with cell behaviors during tissue patterning and morphogenesis, and to determine whether the observed effects are conserved across species. Among other things, it will be interesting and important to establish whether multiple mutations in any given component of the Ras-MAPK pathway can be ranked based on the severity of their biological and biochemical effects on Ras-MAPK-dependent developmental events. In parallel, it is essential to elucidate the mechanistic origins of the effects caused by different mutations. Given that all affected components of the Ras-MAPK pathway are highly regulated, one must explore a range of possibilities, from changes in subcellular localization, to changes in enzymatic activity, to changes in protein-protein interactions. Thus, we are only beginning to understand the mechanistic origins of human RASopathies (Kiel and Serrano, 2014). Elucidating this should help to both address some of the fundamental questions related to the role of the Ras-MAPK pathway during development and provide rational guidelines for the management and treatment of a large class of developmental defects.

## Supplementary Material

Supplementary Material
