# RASopathies: unraveling mechanisms with animal models

**DOI:** 10.1242/dmm.022442

**Published:** 2015-09-01

**Authors:** Granton A. Jindal, Yogesh Goyal, Rebecca D. Burdine, Katherine A. Rauen, Stanislav Y. Shvartsman

There was an error published in *Dis. Model. Mech.* **8**, 769-782.

In Fig. 1B, a blue region spanning residues 33-36 was missing in the *KRAS* gene. The correct figure appears below. There are no changes to the figure legend, which is accurate.

**Fig. 1.**
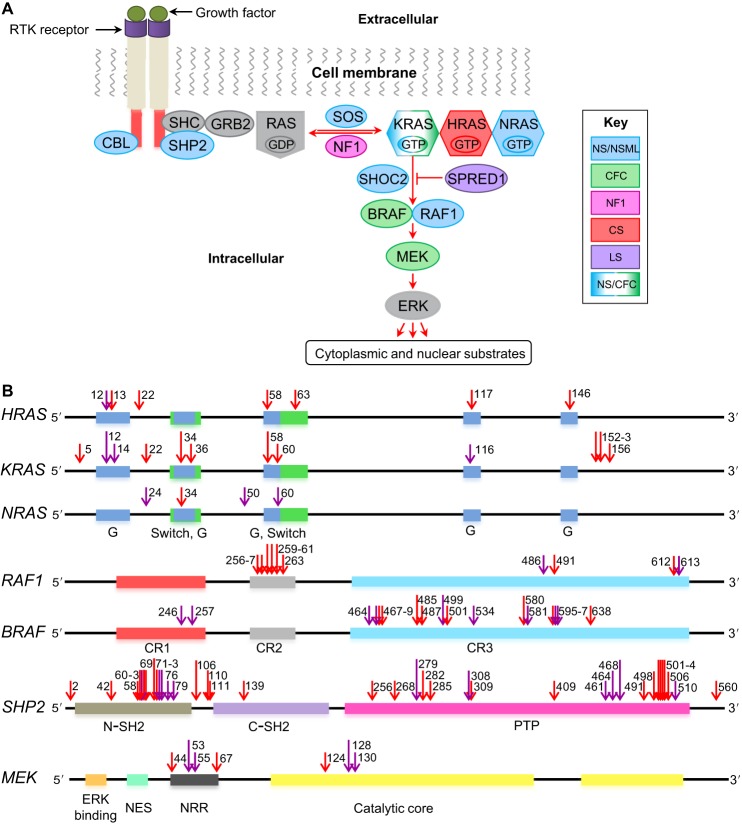
**The Ras-MAPK signaling pathway and associated mutations.** (A) Schematic of the Ras-MAPK signaling pathway. Proteins commonly mutated in RASopathies, color coded to represent different syndromes: Noonan syndrome (NS; blue), cardio-facio-cutaneous syndrome (CFC; green), neurofibromatosis type 1 (NF1; magenta), Costello syndrome (CS; red), Legius syndrome (LS; purple). (B) Positions of mutations in certain genes that encode components of the Ras-MAPK pathway. Purple arrows indicate where a mutation has been modeled in animals; red arrows indicate where it has not. Colored boxes represent regions in the genes that encode key protein domains. In the RAS proteins, the G regions (blue) form the nucleotide-binding site, and the switch regions (green) change conformation between the inactive and active states. In the RAF proteins, the CR1 region (red) contains a Ras-binding domain, the CR2 (gray) and CR3 (turquoise) regions associate with 14-3-3 proteins (a family of key regulatory proteins expressed in all eukaryotic cells). The CR2 region is also a site of regulatory phosphorylation. In the SHP2 protein, structural features include the N (brown) and C (purple) terminal Src homology 2 (SH2) domains, and a protein tyrosine phosphate (PTP) domain (pink). In the MEK protein, key protein domains include the negative regulatory region (NRR; black), the MAPK-binding site (ERK binding; orange), the nuclear export signal (NES; green) and the catalytic core (yellow). Numbers near the arrows indicate the protein residues that are mutated (see supplementary material Table S1 for more details). RTK, receptor tyrosine kinase.

We apologise to the readers and authors for any confusion that this error might have caused.

