# Supplementary material for: RASopathies: unraveling mechanisms with animal models
Source: Dis Model Mech. 2015 Aug 1;8(8):769–82. doi: 10.1242/dmm.020339 (PMC4527292; doi:10.1242/dmm.020339)
Supplement: Supplementary Material [file supp_8_8_769__index.html]

Supplementary Material 

# RASopathies: unraveling mechanisms with animal models

## DMM020339 Supplementary Material

- Supplementary Material
