# Supplementary material for: RASopathies: unraveling mechanisms with animal models
Source: Dis Model Mech. 2015 Aug 1;8(8):769–82. doi: 10.1242/dmm.020339 (PMC4527292; doi:10.1242/dmm.020339)
Supplement: Supplementary Material [file supp_8.8.769_DMM020339supp.pdf]

Table S1: RASopathy mutations in the Ras/MAPK pathway. For each residue that mutations occur at, the reference of an animal model, if made, is given.

SHP2

Point Mutations	Model	Source
T2I		(Aoki et al., 2008)
T42A		(Aoki et al., 2008)
N58K, N58D, N58H		(Aoki et al., 2008)
G60A		(Aoki et al., 2008)
D61N, D61G	Yes	(Araki et al., 2004)
Y62N, Y62D		(Aoki et al., 2008)
Y63C		(Aoki et al., 2008)
E69Q		(Aoki et al., 2008)
F71L, F71I		(Aoki et al., 2008)
A72S, A72G	Yes	(Oishi et al., 2006)
T73I	Yes	(Jopling et al., 2007)
E76D	Yes	(Mohi et al., 2005)
Q79R, Q79P	Yes	(Nakamura et al., 2007)
D106A		(Aoki et al., 2008)
E110A		(Aoki et al., 2008)
E111A		(Aoki et al., 2008)
E139D		(Aoki et al., 2008)
Q256R		(Aoki et al., 2008)
G268S, G268C		(Aoki et al., 2008)
Y279C, Y279S	Yes	(Marin et al., 2011)
I282V		(Aoki et al., 2008)
F285L, F285S, F285C		(Aoki et al., 2008)
N308D, N308S, N308T	Yes	(Araki et al., 2009)
I309V		(Aoki et al., 2008)
G409A		(Aoki et al., 2008)
A461T	Yes	(Jopling et al., 2007)
G464A	Yes	(Jopling et al., 2007)
T468M	Yes	(Tajan et al., 2014)
P491S, P491H, P491L		(Aoki et al., 2008)
R498W		(Aoki et al., 2008)
R501K		(Aoki et al., 2008)
S502T, S502L, S502A		(Aoki et al., 2008)
G503R		(Aoki et al., 2008)
M504V		(Aoki et al., 2008)
Q506P		(Aoki et al., 2008)
Q510E, Q510P	Yes	(Schramm et al., 2012)
L560F		(Aoki et al., 2008)

NRAS

Point Mutations	Model	Source
I24N	Yes	(Runtuwene et al., 2011)
P34L		(Denayer et al., 2012)
T50I	Yes	(Runtuwene et al., 2011)
G60E	Yes	(Runtuwene et al., 2011)

HRAS

Point Mutations	Model	Source
G12S, G12C, G12A, G12V, G12E	Yes	(Schuhmacher et al., 2008)
G13C, G13D		(Sol-Church and Gripp, 2009)
Q22K		(Sol-Church and Gripp, 2009)
T58I		(Sol-Church and Gripp, 2009)
E63K		(Sol-Church and Gripp, 2009)
K117R		(Sol-Church and Gripp, 2009)
A146T, A146V		(Sol-Church and Gripp, 2009)

KRAS

Point Mutations	Model	Source
K5N		(Aoki et al., 2008)
G12D	Yes	(Tuveson et al., 2004)
V14I	Yes	(Hernández-Porras et al., 2014)
Q22E, Q22R		(Aoki et al., 2008)
P34R, P34L, P34Q		(Aoki et al., 2008)
I36M		(Aoki et al., 2008)
T58I		(Aoki et al., 2008)
G60R		(Aoki et al., 2008)
N116S	Yes	(Razzaque et al., 2012)
V152G		(Aoki et al., 2008)
D153V		(Aoki et al., 2008)
F156I, F156L		(Aoki et al., 2008)

MEK

Point Mutations	Model	Source
E44G		(Bromberg-White et al., 2012)
F53S	Yes	(Anastasaki et al., 2009)
T55P	Yes	(Anastasaki et al., 2009)
D67N		(Bromberg-White et al., 2012)
P124Q		(Bromberg-White et al., 2012)
G128V	Yes	(Anastasaki et al., 2009)
Y130C	Yes	(Anastasaki et al., 2009)

BRAF

Point Mutations	Model	Source
A246P	Yes	(Anastasaki et al., 2009)
Q257K, Q257R	Yes	(Anastasaki et al., 2009)
G464V	Yes	(Anastasaki et al., 2009)
S467A	Yes	(Anastasaki et al., 2009)
F468S		(Aoki et al., 2008)
G469E		(Aoki et al., 2008)
L485F		(Aoki et al., 2008)
V487G		(Aoki et al., 2008)
K499E	Yes	(Anastasaki et al., 2009)
E501K, E501G		(Aoki et al., 2008)
G534R	Yes	(Anastasaki et al., 2009)
N580D		(Aoki et al., 2008)
N581D	Yes	(Aoki et al., 2008)
F595L		(Aoki et al., 2008)
G596V	Yes	(Anastasaki et al., 2009)
L597V	Yes	(Andreadi et al., 2012)
D638E		(Aoki et al., 2008)

RAF1

Point Mutations	Model	Source
R256S		(Tartaglia and Gelb, 2009)
S257L		(Tartaglia and Gelb, 2009)
S259F		(Tartaglia and Gelb, 2009)
T260R, T260I		(Tartaglia and Gelb, 2009)
P261S, P261L, P261A		(Tartaglia and Gelb, 2009)
V263A		(Tartaglia and Gelb, 2009)
D486N	Yes	(Wu et al., 2012)
T491I, T491R		(Tartaglia and Gelb, 2009)
S612T		(Tartaglia and Gelb, 2009)
L613V	Yes	(Wu et al., 2011a)

Table S2: Mouse models of NF1

Gene	Defect	Phenotype	Signaling	Reference
Nf1	General	*Nf1*+/-: Predisposition to various tumors, *Nf1*-/-: Mid-gestational embryonic lethality, and cardiac defects such as double outlet right ventricle; hyperplasia of neural crest-derived sympathetic ganglia and delay in renal, hepatic and skeletal muscle development and exencephaly		(Brannan et al., 1994; Ismat et al., 2006; Jacks et al., 1994; Lakkis and Epstein, 1998; Lakkis et al., 1999)
Nf1	Leukemia	Mice can be induced into chronic myeloproliferative disease which is similar to various human leukemias including JCML		(Chang et al., 2013; Gitler et al., 2004; Largaespada et al., 1996)
Nf1	OPG	Complete deletion of *Nf1* in astrocytes and reduced NF1 expression in other cells is required to increase astrocyte proliferation and cause optic pathway gliomas (OPGs). Furthermore, astrocytes differentiating from different germinal zones have different capacities to respond to glioma-causing genetic changes	PI3K/mTOR/Akt pathway	(Bajenaru et al., 2002; Bajenaru et al., 2003; Banerjee et al., 2011a; Banerjee et al., 2011b; Dahiya et al., 2011; Dasgupta et al., 2005a; Dasgupta et al., 2005b; Hegedus et al., 2007; Lee et al., 2010a; Lee et al., 2012; Solga et al., 2014; Yeh et al., 2009; Zhu et al., 2001; Zhu et al., 2005)
Nf1	OPG	Neuronal support cells, microglia, are needed for astrocyte proliferation and OPG formation		(Daginakatte and Gutmann, 2007; Daginakatte et al., 2008; Pong et al., 2013; Simmons et al., 2011; Sun et al., 2010; Warrington et al., 2007; Warrington et al., 2010)
Nf1	Neurofib-roma	Dermal, plexiform neurofibromas and Malignant Peripheral Nerve Sheath Tumor (MPNST)		(Cichowski et al., 1999; Jessen et al., 2013; Le et al., 2009; Mayes et al., 2011; Reilly et al., 2006; Rosenbaum et al., 1997; Vogel et al., 1999; Wu et al., 2008; Yang et al., 2008; Zhu et al., 2002)
Nf1	Bone	*Nf1*+/-: less bone formation and impaired healing of distal tibial fractures , tissue specific *Nf1*-/-: bowing of the tibia (*Prx1*), congenital limb shortening (*Prx1*), defective fracture healing (*Prx1*, *Col2.3*, *Osx1*), reduced muscle fibers and strength (*Prx1*), abnormal development of joints (*Prx1*), reduced bone mass (*Col2.3*) , short stature (*Prx1* and *Col2*), impaired bone strength, short vertebral segments (*Col2.3*), altered muscle metabolism (*Prx1* and muscle)	Increased TGF-β1 signaling, increased pyrophosphates, impaired mitochondrial activity	(de la Croix Ndong et al., 2014; de la Croix Ndong et al., 2015; El Khassawna et al., 2012; Kossler et al., 2011; Rhodes et al., 2013; Schindeler et al., 2008; Wang et al., 2011; Wu et al., 2011b; Zhang et al., 2011)
Nf1	Behavior	Motivational disturbances, spatial learning defects, attention defects, working memory defects, motor learning defects, social learning defects, sex-specific learning defects	Ras/MAPK pathway and dopamine homeostasis	(Brown et al., 2010; Costa et al., 2001; Costa et al., 2002; Cui et al., 2008; Diggs-Andrews et al., 2013; Diggs-Andrews et al., 2014; Guilding et al., 2007; Li et al., 2005; Shilyansky et al., 2010; Silva et al., 1997; van der Vaart et al., 2011; Wozniak et al., 2013)
Nf1	Brain	Increase in size of cerebral cortical surface area, increased thickness of corpus callosum	Ras/MAPK pathway	(Wang et al., 2012)
Nf1	Heart defects	Mice display Ras/MAPK-dependent defects in endocardial cushions which obstructs blood flow. Embryos with endothelial cell-specific biallelic deletion have defects in endocardial cushions and myocardium, while embryos with myocardial cell-specific biallelic deletion display some adult-onset heart defects.	Ras/MAPK pathway	(Gitler et al., 2003; Ismat et al., 2006; Lakkis and Epstein, 1998; Lasater et al., 2008; Lasater et al., 2010; Xu et al., 2007; Xu et al., 2009)

Table S3: Mouse models of non-NF1 RASopathies [1]Cre-lox [2]Tissue-specific Cre-lox [3]Overexpression [4]Other

Gene	Protein (Function)	Mutation	Disease	Phenotype	Signaling	Reference
Ptpn11	Shp2 (Phosphatase)	D61G[1]	NS	Hom: embryonic lethal; Het: Short stature, craniofacial defects, and myeloproliferative diease (MPD). Severely affected embryos with cardiac defects; Increased angiogenesis; Neurocognitive defects	Increased Ras/MAPK, short stature by IGF-1	(Araki et al., 2004; De Rocca Serra-Nédélec et al., 2012; Lee et al., 2014; Wang et al., 2009; Xu et al., 2010)
Ptpn11	Shp2 (Phosphatase)	D61Y/G, E76K[1,4]	NS	Impaired hematopoiesis, increased reactive oxygen species leading to MPD		(Mohi et al., 2005; Xu et al., 2013)
Ptpn11	Shp2 (Phosphatase)	Q79R[2]	NS	Heart defects and enlarged endocardial cushions; craniofacial defects including smaller skull lengths and taller frontal bone heights; Elevated oligodendrocyte precursor cell (OPC) numbers	Increased Ras/MAPK in heart, frontal bones, and brain	(Ehrman et al., 2014; Krenz et al., 2008; Nakamura et al., 2007; Nakamura et al., 2009)
Ptpn11	Shp2 (Phosphatase)	N308D, D61Y[1,2]	NS	D61Y Het, N308D Hom: Cardiac, growth, facial, and hematologic defects similar to D61G Het; N308D Het: Less severe than N308D Hom	Increased Ras/MAPK	(Araki et al., 2009)
Ptpn11	Shp2 (Phosphatase)	Y279C[1]	NSML	Het: Short stature, craniofacial dysmorphia, hypertrophic cardiomyopathy (HCM), and increased heart to body weight ratio.	Increased AKT/mTOR	(Marin et al., 2011)
Ptpn11	Shp2 (Phosphatase)	Q510E[3]	NSML	Increased cardiomyocyte sizes, heart-to-body weight ratios, interventricular septum thickness, and cardiomyocyte disarray.	Increased mTOR	(Edwards et al., 2015; Schramm et al., 2012)
Ptpn11	Shp2 (Phosphatase)	T468M[1]	NSML	Impaired adipogenesis and better metabolic profile resulting in lean mice, splenomegaly, and larger heart.	Increased AKT and Ras/MAPK	(Tajan et al., 2014)
Sos1	Sos1 (Ras GEF)	E846K[1]	NS	Growth delay, distinctive facial dysmorphia, hematologic abnormalities, and cardiac defects.	Increased Ras/MAPK, Rac, and Stat3	(Chen et al., 2010)
Raf1	Kinase	L613V[1]	NS	Het: Short stature, craniofacial dysmorphia, splenomegaly, hematologic defects, and HCM.	Increased Ras/MAPK	(Marin et al., 2011)
Raf1	Kinase	D486N[1]	NS	Het: female mice exhibited a mild growth defect; Hom: heart defects and incompletely penetrant, but severe, growth defects.	Increased Ras/MAPK in vitro	(Wu et al., 2012)
Braf	Kinase	V600E[1]	CFC	Reduced size and body weight, craniofacial defects, cardiomegaly, and epileptic seizures	No change in Ras/MAPK	(Urosevic et al., 2011)
Braf	Kinase	L597V[1]	CFC	Short stature, craniofacial defects, HCM, cardiac enlargement, and skin papillomas	Increased Ras/MAPK	(Andreadi et al., 2012)
Braf	GTPase	Q241R[1]	CFC	Embryonic lethal, embryonic skeletal abnormalities, lymphatic defects, and cardiac defects	Increased Ras/MAPK in brain, decreased p38 & Akt	(Inoue et al., 2014)
Kras	GTPase	G12D[1,2]	NS	Small and pale embryos with brain defects and cardiomegaly		(Tuveson et al., 2004)
Kras	GTPase	V14I[1]	NS	Het: Growth delay, craniofacial defects, cardiac defects, and hematologic defects (MPD); Hom: perinatal lethality	Increased Ras/MAPK	(Hernández-Porras et al., 2014)
Kras	GTPase	G12D[2]	CFC, NS	No changes in heart size or contractility	Increased Ras/MAPK in tissue	(Dalin et al., 2014)
Hras	GTPase	G12V[1]	CS	Het: Viable but have facial dysmorphia, HCM, and hypertension; Hom: Same phenotypes but more penetrant with hyperemotivity, hypersensitivity, and cognitive impairments	No change in Ras/MAPK and Akt	(Schuhmacher et al., 2008; Viosca et al., 2009)
Hras	GTPase	G12V[1]	CS	High perinatal lethality, craniofacial defects including nasal septal deviation, teeth defects, high penetrant neoplastic phenotypes, skin papillomas under certain conditions	No change in Ras/MAPK and Akt except liver (increased Ras/MAPK)	(Chen et al., 2009; Chen et al., 2014; Goodwin et al., 2014)
Hras	GTPase	G12V[4]	CS	Increased hepatocyte growth leading to hepatomegaly		(Figueiredo et al., 2012)

Table S4: Zebrafish Models of RASopathies [1]MO injections, [2]Zinc finger nucleases, [3]mRNA Injection, [4]Transposon-mediated integration & Heat Shock, [5]Injection of vector

Gene	Protein (Function)	Mutation	Disease	Phenotype	Signaling	Reference
nf1	Neurofibromin (Ras GAP)	Knockout[1]	NF1	Cardiac malformations and defects in vascular patterning, OPC hyperplasia, and OPC migration.	Increased Ras/MAPK	(Lee et al., 2010b; Padmanabhan et al., 2009)
nf1	Neurofibromin (Ras GAP)	Knockout[2]	NF1	OPC and Schwann cell hyperplasia, aberrant myelination, motor and learning defects, and melanophore defects.	Increased Ras/MAPK	(Shin et al., 2012; Zhu et al., 2011)
nf1	Neurofibromin (Ras GAP)	Knockout[2]	NF1	Learning and memory deficits.	Increased Ras/MAPK, PI3K, and cAMP	(Wolman et al., 2014)
ptpn11	Shp2 (Phosphatase)	D61G, T73I, A462T, G465A[3]	NS / NSML	Craniofacial defects, pericardial edema, heart jogging defects, convergence and extension (CE) defects, and shorter larvae.		(Jopling et al., 2007)
ptpn11	Shp2 (Phosphatase)	A462T[3]	NSML	Craniofacial dysmorphia, severe gastrulation phenotypes, a hammerhead phenotype, or no obvious phenotype. It alters neural crest specification and migration.	Shp2 is upstream of Ras/MAPK and Sh2 domain-dependent pathway to prevent cell death	(Stewart et al., 2010)
ptpn11	Shp2 (Phosphatase)	T468M[3]	NSML	Pericardial edema to varying degrees.		(Miura et al., 2013)
ptpn11	Shp2 (Phosphatase)	D61G, T73I, A462T, G465A[3]	NS / NSML	Defects in left-right heart jogging. These are tied to defects in cilia, Kupffer's vesicle and the propagation of Nodal in the lateral plate mesoderm.	Increased Ras/MAPK	(Bonetti et al., 2014)
ptpn11	Shp2 (Phosphatase)	Y279C, T468M	NSML	Convergence and extension defects are measured by the ratio of rhombomere width over rhombomere length.	PZR downstream of Shp2	(Overman et al., 2014)
hras	GTPase	G12V[4]	CS	Tumors, reduced size and life span, smaller heart, and craniofacial defects in adult fish.	Still largely unknown	(Santoriello et al., 2009)
nras	GTPase	I24N, T50I, G60E, G12V[3]	NS	Craniofacial defects and CE defects which cause an oblong embryo shape.	Increased Ras/MAPK	(Runtuwene et al., 2011)
kras	GTPase	N116S[3]	NS	Craniofacial dysmorphia, reduced heart size, and heart jogging defects.	Increased Ras/MAPK	(Razzaque et al., 2012)
braf/ mek	Kinase	28 mutations tested[3]	CFC	CE defects which result in an oblong embryo shape. Heart defects are reported.	Increased Ras/MAPK	(Anastasaki et al., 2009)
braf	Kinase	Q257R, G596V[3]	CFC	Similar to 2009 paper except they find that heart is sensitive to signaling perturbations.	Increased Ras/MAPK	(Anastasaki et al., 2012)
a2ml1	a-2-macroglob ulin (A2M)-like-1 (Protease inhibitor)	S592L, R802H, R802L[5]	NS	Craniofacial defects scored by width over height ratio of head and heart looping.	No change in Ras/MAPK	(Vissers et al., 2015)
rit1	RIT1 (GTPase)	Q79L, E81G, G95A[3]	NS	CE defects cause an oblong embryo shape. There are craniofacial and heart defects.	Increased RIT1/MAPK	(Aoki et al., 2013)

Table S5: *Drosophila* Models of RASopathies [1]P-element insertion at NF1 locus, [2]Chemical mutagenesis, [3]Overexpression using p-element insertion

Gene	Protein (function)	Mutation	Disease	Phenotype	Signaling	Reference
Nf1	Neurofibromin (Ras GAP)	Knockout[1]	NF1	$NF1^{-/-}$ flies have growth defects throughout development and impaired cellular response to PACAP38, a neuropeptide	Impaired cAMP	(Guo et al., 1997; The et al., 1997; Tong et al., 2002)
Nf1	Neurofibromin (Ras GAP)	Knockout[1]	NF1	Olfactory learning and short term memory defects	Impaired cAMP	(Guo et al., 2000)
Nf1	Neurofibromin (Ras GAP)	Knockout[1]	NF1	Abnormal circadian rhythms	Increased Ras/MAPK	(Williams et al., 2001)
Nf1	Neurofibromin (Ras GAP)	Knockout[2]	NF1	Growth defects throughout development	Increased Ras/MAPK	(Walker et al., 2006)
Nf1	Neurofibromin (Ras GAP)	Knockout[1]	NF1		Novel NF1-Ras-AC signaling, independent of G_{α} subunit.	(Hannan et al., 2006)
Nf1	Neurofibromin (Ras GAP)	Knockout[1]	NF1	Shortened life expectancy, vulnerability to oxidative and heat stresses, reduced mitochondrial respiration and elevated reactive oxygen species (ROS) production	cAMP regulates mitochondrial activity	(Tong et al., 2007)
Nf1	Neurofibromin (Ras GAP)	Knockout[1]	NF1	Learning and long term memory (LTM) defects	GAP-related domain needed for LTM and C-terminal essential for short- and intermediate-term memory	(Buchanan and Davis, 2010; Ho et al., 2007)
Nf1	Neurofibromin (Ras GAP)	Knockout[2]	NF1		Alk and other modifier genes involved in NF1	(Gouzi et al., 2011; Walker et al., 2013)
csw	Shp-2 (phosphatase)	D61Y, E76K[3]	NS	LTM defects	Prolonged activation or slow decay of Ras/MAPK cycle	(Pagani et al., 2009)
csw	Shp-2 (phosphatase)	N308D, A72S, E76K[3]	NS	Ectopic veins	Increased Ras/MAPK	(Oishi et al., 2006)
csw	Shp-2 (phosphatase)	Y279C,T468M[3]	NSML	Ectopic veins, rough eye	Increased Ras/MAPK	(Oishi et al., 2009)
csw	Shp-2 (phosphatase)	E76K[3]	Leukemia	Increased plasmatocyte cells	Increased Ras/MAPK	(Mohi et al., 2005)
Ras	GTPase	R68Q[2]	NS/CS/ CFC	Enhanced resistance to cell death, ectopic veins, rough eye	Increased Ras/MAPK	(Gafuik and Steller, 2011)
Ras	GTPase	G12V[3]	NS	Cardiac hypertrophy	Increased Ras/MAPK	(Yu et al., 2013)

Table S6: Phenotypes of Animal Models of RASopathies. For each phenotype that occurs in each RASopathy, a reference of an animal model is given.

Diagram Part	Phenotypes	RASopathy	Reference
**Mice Only**	Bone defects	NF1	(Kolanczyk et al., 2007)
	Neurofibromas	NF1	(Rosenbaum et al., 1997)
	Sex-linked effects	NF1	(Diggs-Andrews et al., 2014)
	Muscle Abnormalities	NF1	(Sullivan et al., 2014)
	Attention deficits	NF1	(Brown et al., 2010)
	Working memory deficits	NF1	(Shilyansky et al., 2010)
	Leaner metabolic profile	NS	(Tajan et al., 2014)
	Hematologic disease	NS	(Araki et al., 2009)
	Triangular face	NS, CS	(Araki et al., 2004; Schuhmacher et al., 2008)
	Enlarged spleen	NS	(Araki et al., 2004)
	Liver defects	NF1,NS,CFC,CS	(Araki et al., 2004; Figueiredo et al., 2012; Hegedus et al., 2007; Inoue et al., 2014)
	Lymphatic system defects	CFC	(Inoue et al., 2014)
	Epileptic seizures	CFC	(Urosevic et al., 2011)
	Nasal septal deviation	CS	(Chen et al., 2009)
	Papilloma formation	CS	(Chen et al., 2009)
	Hyperemotivity	CS	(Viosca et al., 2009)
	Teeth defects	CS	(Goodwin et al., 2014)

Diagram Part	Phenotypes	RASopathy	Reference
***Drosophila* Only**	Mitochondrial defects	NF1	(Tong et al., 2007)
	Circadian rhythm defects	NF1	(Williams et al., 2001)
	Synaptic overgrowth	NF1	(Walker et al., 2013)
	Slower escape response	NF1	(The et al., 1997)
	Ectopic veins	NS	(Oishi et al., 2006)
	Photoreceptor defects	NS	(Oishi et al., 2006)
**Zebrafish Only**	Pigmentation Defects	NF1	(Shin et al., 2012)
	Motor Defects	NF1	(Shin et al., 2012)
	Schwann cell hyperplasia	NF1	(Shin et al., 2012)
	Kupffer's vesicle malformation	NS	(Bonetti et al., 2014)
	C&E defects	NS, CFC	(Runtuwene et al., 2011)
	Precocious ossification	CS	(Santoriello et al., 2009)
	Reduced blood oxygenation	CS	(Santoriello et al., 2009)
	Scoliotic spine	CS	(Santoriello et al., 2009)
	Sterility	CS	(Santoriello et al., 2009)

Diagram Part	Phenotypes	RASopathy	Reference	RASopathy	Reference	RASopathy	Reference
**Mice and *Drosophila***		**Mice**		***Drosophila***			
	Myeloproliferative disease	NF1, NS	(Gitler et al., 2004; Mohi et al., 2005)	NS	(Mohi et al., 2005)		
**Mice and Zebrafish**		**Mice**		**Zebrafish**			
	Neural crest cell defects	NF1	(Ismat et al., 2006)	NF1, NS	(Shin et al., 2012; Stewart et al., 2010)		
	Myelin sheath defects	NF1	(Cichowski et al., 1999)	NF1	(Shin et al., 2012)		
	OPC hyperplasia	NF1, NS	(Bennett et al., 2003; Ehrman et al., 2014)	NF1	(Shin et al., 2012)		
	Hypertelorism	NS	(Araki et al., 2004)	NS, CFC, CS	(Anastasaki et al., 2012; Runtuwene et al., 2011; Santoriello et al., 2009)		
	Gliomas	NF1	(Hegedus et al., 2009)	NF1	(Shin et al., 2012)		
**Mice, Zebrafish, and *Drosophila***		**Mouse**		**Zebrafish**		***Drosophila***	
	Learning/cognitive defects	NF1, NS, CS	(Costa et al., 2002; Lee et al., 2014; Viosca et al., 2009)	NF1	(Wolman et al., 2014)	NF1, NS	(Buchanan and Davis, 2010; Pagani et al., 2009)
	Reduced life span	NS, CFC	(Hernández-Porras et al., 2014; Urosevic et al., 2011)	CS	(Santoriello et al., 2009)	NF1	(Tong et al., 2007)
	Growth defects	NS, CFC	(Araki et al., 2004; Urosevic et al., 2011)	NS, CFC, CS	(Anastasaki et al., 2009; Jopling et al., 2007; Santoriello et al., 2009)	NF1	(Walker et al., 2006)
	Cardiac defects	NF1, NS, CFC, CS	(Araki et al., 2009; Inoue et al., 2014; Ismat et al., 2006; Schuhmacher et al., 2008)	NF1, NS, CS	(Bonetti et al., 2014; Padmanabhan et al., 2009; Santoriello et al., 2009)	NS	(Yu et al., 2013)

References

Anastasaki, C., Estep, A. L., Marais, R., Rauen, K. A. and Patton, E. E. (2009). Kinase-activating and kinase-impaired cardio-facio-cutaneous syndrome alleles have activity during zebrafish development and are sensitive to small molecule inhibitors. *Hum. Mol. Genet.* **18**, 2543–54.

Anastasaki, C., Rauen, K. A. and Patton, E. E. (2012). Continual low-level MEK inhibition ameliorates cardio-facio-cutaneous phenotypes in zebrafish. *Dis. Model. Mech.* **5**, 546–52.

Andreadi, C., Cheung, L.-K., Giblett, S., Patel, B., Jin, H., Mercer, K., Kamata, T., Lee, P., Williams, A., McMahon, M., et al. (2012). The intermediate-activity (L597V)BRAF mutant acts as an epistatic modifier of oncogenic RAS by enhancing signaling through the RAF/MEK/ERK pathway. *Genes Dev.* **26**, 1945–58.

Aoki, Y., Niihori, T., Narumi, Y., Kure, S. and Matsubara, Y. (2008). The RAS/MAPK syndromes: novel roles of the RAS pathway in human genetic disorders. *Hum. Mutat.* **29**, 992–1006.

Aoki, Y., Niihori, T., Banjo, T., Okamoto, N., Mizuno, S., Kurosawa, K., Ogata, T., Takada, F., Yano, M., Ando, T., et al. (2013). Gain-of-function mutations in RIT1 cause Noonan syndrome, a RAS/MAPK pathway syndrome. *Am. J. Hum. Genet.* **93**, 173–80.

Araki, T., Mohi, M. G., Ismat, F. A., Bronson, R. T., Williams, I. R., Kutok, J. L., Yang, W., Pao, L. I., Gilliland, D. G., Epstein, J. A., et al. (2004). Mouse model of Noonan syndrome reveals cell type- and gene dosage-dependent effects of Ptpn11 mutation. *Nat. Med.* **10**, 849–57.

Araki, T., Chan, G., Newbigging, S., Morikawa, L., Bronson, R. T. and Neel, B. G. (2009). Noonan syndrome cardiac defects are caused by PTPN11 acting in endocardium to enhance endocardial-mesenchymal transformation. *Proc. Natl. Acad. Sci. U. S. A.* **106**, 4736–41.

Bajenaru, M. L., Zhu, Y., Hedrick, N. M., Donahoe, J., Parada, L. F. and Gutmann, D. H. (2002). Astrocyte-specific inactivation of the neurofibromatosis 1 gene (NF1) is insufficient for astrocytoma formation. *Mol. Cell. Biol.* **22**, 5100–13.

Bajenaru, M. L., Hernandez, M. R., Perry, A., Zhu, Y., Parada, L. F., Garbow, J. R. and Gutmann, D. H. (2003). Optic nerve glioma in mice requires astrocyte Nf1 gene inactivation and Nf1 brain heterozygosity. *Cancer Res.* **63**, 8573–7.

Banerjee, S., Crouse, N. R., Emnett, R. J., Gianino, S. M. and Gutmann, D. H. (2011a). Neurofibromatosis-1 regulates mTOR-mediated astrocyte growth and glioma formation in a TSC/Rheb-independent manner. *Proc. Natl. Acad. Sci. U. S. A.* **108**, 15996–16001.

Banerjee, S., Gianino, S. M., Gao, F., Christians, U. and Gutmann, D. H. (2011b). Interpreting mammalian target of rapamycin and cell growth inhibition in a genetically engineered mouse model of Nf1-deficient astrocytes. *Mol. Cancer Ther.* **10**, 279–91.

Bennett, M. R., Rizvi, T. A., Karyala, S., McKinnon, R. D. and Ratner, N. (2003). Aberrant growth and differentiation of oligodendrocyte progenitors in neurofibromatosis type 1 mutants. *J. Neurosci.* **23**, 7207–17.

Bonetti, M., Paardekooper Overman, J., Tessadori, F., Noël, E., Bakkers, J. and den Hertog, J. (2014). Noonan and LEOPARD syndrome Shp2 variants induce heart displacement defects in zebrafish. *Development* **141**, 1961–70.

Brannan, C. I., Perkins, A. S., Vogel, K. S., Ratner, N., Nordlund, M. L., Reid, S. W., Buchberg, A. M., Jenkins, N. A., Parada, L. F. and Copeland, N. G. (1994). Targeted disruption of the neurofibromatosis type-1 gene leads to developmental abnormalities in heart and various neural crest-derived tissues. *Genes Dev.* **8**, 1019–29.

Bromberg-White, J. L., Andersen, N. J. and Duesbery, N. S. (2012). MEK genomics in development and disease. *Brief. Funct. Genomics* **11**, 300–10.

Brown, J. A., Emnett, R. J., White, C. R., Yuede, C. M., Conyers, S. B., O'Malley, K. L., Wozniak, D. F. and Gutmann, D. H. (2010). Reduced striatal dopamine underlies the attention system dysfunction in neurofibromatosis-1 mutant mice. *Hum. Mol. Genet.* **19**, 4515–28.

Buchanan, M. E. and Davis, R. L. (2010). A Distinct Set of Drosophila Brain Neurons Required for Neurofibromatosis Type 1-Dependent Learning and Memory. *J. Neurosci.* **30**, 10135–10143.

Chang, T., Krisman, K., Theobald, E. H., Xu, J., Akutagawa, J., Lauchle, J. O., Kogan, S., Braun, B. S. and Shannon, K. (2013). Sustained MEK inhibition abrogates myeloproliferative disease in Nf1 mutant mice. *J. Clin. Invest.* **123**, 335–9.

Chen, X., Mitsutake, N., LaPerle, K., Akeno, N., Zanzonico, P., Longo, V. A., Mitsutake, S., Kimura, E. T., Geiger, H., Santos, E., et al. (2009). Endogenous expression of Hras(G12V) induces developmental defects and neoplasms with copy number imbalances of the oncogene. *Proc. Natl. Acad. Sci. U. S. A.* **106**, 7979–84.

Chen, P., Wakimoto, H., Conner, D., Araki, T., Yuan, T., Roberts, A., Seidman, C. E., Bronson, R., Neel, B. G., Seidman, J. G., et al. (2010). Activation of multiple signaling pathways causes developmental defects in mice with a Noonan syndrome – associated Sos1 mutation. *J. Clin. Invest.* **120**, 4353–4365.

Chen, X., Makarewicz, J. M., Knauf, J. A., Johnson, L. K. and Fagin, J. A. (2014). Transformation by Hras(G12V) is consistently associated with mutant allele copy gains and is reversed by farnesyl transferase inhibition. *Oncogene* **33**, 5442–5449.

Cichowski, K., Shih, T. S., Schmitt, E., Santiago, S., Reilly, K., McLaughlin, M. E., Bronson, R. T. and Jacks, T. (1999). Mouse models of tumor development in neurofibromatosis type 1. *Science* **286**, 2172–6.

Costa, R. M., Yang, T., Huynh, D. P., Pulst, S. M., Viskochil, D. H., Silva, A. J. and Brannan, C. I. (2001). Learning deficits, but normal development and tumor predisposition, in mice lacking exon 23a of Nf1. *Nat. Genet.* **27**, 399–405.

Costa, R. M., Federov, N. B., Kogan, J. H., Murphy, G. G., Stern, J., Ohno, M., Kucherlapati, R., Jacks, T. and Silva, A. J. (2002). Mechanism for the learning deficits in a mouse model of neurofibromatosis type 1. *Nature* **415**, 526–30.

Cui, Y., Costa, R. M., Murphy, G. G., Elgersma, Y., Zhu, Y., Gutmann, D. H., Parada, L. F., Mody, I. and Silva, A. J. (2008). Neurofibromin regulation of ERK signaling modulates GABA release and learning. *Cell* **135**, 549–60.

Daginakatte, G. C. and Gutmann, D. H. (2007). Neurofibromatosis-1 (Nf1) heterozygous brain microglia elaborate paracrine factors that promote Nf1-deficient astrocyte and glioma growth. *Hum. Mol. Genet.* **16**, 1098–112.

Daginakatte, G. C., Gianino, S. M., Zhao, N. W., Parsadanian, A. S. and Gutmann, D. H. (2008). Increased c-Jun-NH2-kinase signaling in neurofibromatosis-1 heterozygous microglia drives microglia activation and promotes optic glioma proliferation. *Cancer Res.* **68**, 10358–66.

Dahiya, S., Lee, D. Y. and Gutmann, D. H. (2011). Comparative Characterization of the Human and Mouse Third Ventricle Germinal Zones. *J Neuropathol Exp Neurol.* **70**, 622–633.

Dalin, M. G., Zou, Z., Scharin-Täng, M., Safari, R., Karlsson, C. and Bergo, M. O. (2014). Myocardial KRAS(G12D) expression does not cause cardiomyopathy in mice. *Cardiovasc. Res.* **101**, 229–35.

Dasgupta, B., Li, W., Perry, A. and Gutmann, D. H. (2005a). Glioma Formation in Neurofibromatosis 1 Reflects Preferential Activation of K-RAS in Astrocytes. *Cancer Res.* **65**, 236–245.

Dasgupta, B., Yi, Y., Chen, D. Y., Weber, J. D. and Gutmann, D. H. (2005b). Proteomic Analysis Reveals Hyperactivation of the Mammalian Target of Rapamycin Pathway in Neurofibromatosis 1 − Associated Human and Mouse Brain Tumors. *Cancer Res.* **65**, 2755–2760.

De la Croix Ndong, J., Makowski, A. J., Uppuganti, S., Vignaux, G., Ono, K., Perrien, D. S., Joubert, S., Baglio, S. R., Granchi, D., Stevenson, D. A., et al. (2014). Asfotase-α improves bone growth, mineralization and strength in mouse models of neurofibromatosis type-1. *Nat. Med.* **20**, 904–10.

De la Croix Ndong, J., Stevens, D. M., Vignaux, G., Uppuganti, S., Perrien, D. S., Yang, X., Nyman, J. S., Harth, E. and Elefteriou, F. (2015). Combined MEK inhibition and BMP2 treatment promotes osteoblast differentiation and bone healing in Nf1OSX (-/-) mice. *J. bone Miner. Res.* **30**, 55–63.

De Rocca Serra-Nédélec, A., Edouard, T., Tréguer, K., Tajan, M., Araki, T., Dance, M., Mus, M., Montagner, A., Tauber, M., Salles, J.-P., et al. (2012). Noonan syndrome-causing SHP2 mutants inhibit insulin-like growth factor 1 release via growth hormone-induced ERK hyperactivation, which contributes to short stature. *Proc. Natl. Acad. Sci. U. S. A.* **109**, 4257–62.

Denayer, E., Peeters, H., Sevenants, L., Derbent, M., Fryns, J. P. and Legius, E. (2012). NRAS Mutations in Noonan Syndrome. *Mol. Syndromol.* 34–38.

Diggs-Andrews, K. A., Tokuda, K., Izumi, Y., Zorumski, C. F., Wozniak, D. F. and Gutmann, D. H. (2013). Dopamine deficiency underlies learning deficits in neurofibromatosis-1 mice. *Ann. Neurol.* **73**, 309–15.

Diggs-Andrews, K. A., Brown, J. A., Gianino, S. M., Rubin, J. B., Wozniak, D. F. and Gutmann, D. H. (2014). Sex Is a major determinant of neuronal dysfunction in neurofibromatosis type 1. *Ann. Neurol.* **75**, 309–16.

Edwards, M. A., Crombie, K., Schramm, C. and Krenz, M. (2015). The Q510E mutation in Shp2 perturbs heart valve development by increasing cell migration. *J. Appl. Physiol.* **118**, 124–31.

Ehrman, L. A., Nardini, D., Ehrman, S., Rizvi, T. A., Gulick, J., Krenz, M., Dasgupta, B., Robbins, J., Ratner, N., Nakafuku, M., et al. (2014). The Protein Tyrosine Phosphatase Shp2 Is Required for the Generation of Oligodendrocyte Progenitor Cells and Myelination in the Mouse Telencephalon. *J. Neurosci.* **34**, 3767–3778.

El Khassawna, T., Toben, D., Kolanczyk, M., Schmidt-Bleek, K., Koennecke, I., Schell, H., Mundlos, S. and Duda, G. N. (2012). Deterioration of fracture healing in the mouse model of NF1 long bone dysplasia. *Bone* **51**, 651–60.

Figueiredo, M. L., Stein, T. J., Jochem, A. and Sandgren, E. P. (2012). Mutant Hras(G12V) and Kras(G12D) have overlapping, but non-identical effects on hepatocyte growth and transformation frequency in transgenic mice. *Liver Int.* **32**, 582–91.

Gafuik, C. and Steller, H. (2011). A gain-of-function germline mutation in Drosophila ras1 affects apoptosis and cell fate during development. *PLoS One* **6**, e23535.

Gitler, A. D., Zhu, Y., Ismat, F. A., Lu, M. M., Yamauchi, Y., Parada, L. F. and Epstein, J. A. (2003). Nf1 has an essential role in endothelial cells. *Nat. Genet.* **33**, 75–9.

Gitler, A. D., Kong, Y., Choi, J. K., Zhu, Y., Pear, W. S. and Epstein, J. a (2004). Tie2-Cre-induced inactivation of a conditional mutant Nf1 allele in mouse results in a myeloproliferative disorder that models juvenile myelomonocytic leukemia. *Pediatr. Res.* **55**, 581–4.

Goodwin, A. F., Tidyman, W. E., Jheon, A. H., Sharir, A., Zheng, X., Charles, C., Fagin, J. A., McMahon, M., Diekwisch, T. G. H., Ganss, B., et al. (2014). Abnormal Ras signaling in Costello syndrome (CS) negatively regulates enamel formation. *Hum. Mol. Genet.* **23**, 682–92.

Gouzi, J. Y., Moressis, A., Walker, J. A., Apostolopoulou, A. A., Palmer, R. H., Bernards, A. and Skoulakis, E. M. C. (2011). The receptor tyrosine kinase Alk controls neurofibromin functions in Drosophila growth and learning. *PLoS Genet.* **7**, e1002281.

Guilding, C., McNair, K., Stone, T. W. and Morris, B. J. (2007). Restored plasticity in a mouse model of neurofibromatosis type 1 via inhibition of hyperactive ERK and CREB. *Eur. J. Neurosci.* **25**, 99–105.

Guo, H. F., The, I., Hannan, F., Bernards, A. and Zhong, Y. (1997). Requirement of Drosophila NF1 for activation of adenylyl cyclase by PACAP38-like neuropeptides. *Science* **276**, 795–8.

Guo, H. F., Tong, J., Hannan, F., Luo, L. and Zhong, Y. (2000). A neurofibromatosis-1-regulated pathway is required for learning in Drosophila. *Nature* **403**, 895–8.

Hannan, F., Ho, I., Tong, J. J., Zhu, Y., Nurnberg, P. and Zhong, Y. (2006). Effect of neurofibromatosis type I mutations on a novel pathway for adenylyl cyclase activation requiring neurofibromin and Ras. *Hum. Mol. Genet.* **15**, 1087–98.

Hegedus, B., Dasgupta, B., Shin, J. E., Emnett, R. J., Hart-Mahon, E. K., Elghazi, L., Bernal-Mizrachi, E. and Gutmann, D. H. (2007). Neurofibromatosis-1 regulates neuronal and glial cell differentiation from neuroglial progenitors in vivo by both cAMP- and Ras-dependent mechanisms. *Cell Stem Cell* **1**, 443–57.

Hegedus, B., Hughes, W. F., Garbow, J. R., Gianino, S., Banerjee, D., Kim, K., Ellisman, M. H., Milam, A., Jr, B. and Gutmann, D. H. (2009). Optic Nerve Dysfunction in a Mouse Model of Neurofibromatosis-1 Optic Glioma. *J Neuropathol Exp Neurol.* **68**, 542–551.

Hernández-Porras, I., Fabbiano, S., Schuhmacher, A. J., Aicher, A., Cañamero, M., Cámara, J. A., Cussó, L., Desco, M., Heeschen, C., Mulero, F., et al. (2014). K-RasV14I recapitulates Noonan syndrome in mice. *Proc. Natl. Acad. Sci. U. S. A.* **111**, 16395–400.

Ho, I. S., Hannan, F., Guo, H.-F., Hakker, I. and Zhong, Y. (2007). Distinct functional domains of neurofibromatosis type 1 regulate immediate versus long-term memory formation. *J. Neurosci.* **27**, 6852–7.

Inoue, S.-I., Moriya, M., Watanabe, Y., Miyagawa-Tomita, S., Niihori, T., Oba, D., Ono, M., Kure, S., Ogura, T., Matsubara, Y., et al. (2014). New BRAF knockin mice provide a pathogenetic mechanism of developmental defects and a therapeutic approach in cardio-facio-cutaneous syndrome. *Hum. Mol. Genet.* **23**, 6553–66.

Ismat, F. A., Xu, J., Lu, M. M. and Epstein, J. A. (2006). The neurofibromin GAP-related domain rescues endothelial but not neural crest development in Nf1 –/– mice. *J. Clin. Invest.* **116**, 2378–2384.

Jacks, T., Shih, T. S., Schmitt, E. M., Bronson, R. T., Bernards, A. and Weinberg, R. A. (1994). Tumour predisposition in mice heterozygous for a targeted mutation in Nf1. *Nat. Genet.* **7**, 353–61.

Jessen, W. J., Miller, S. J., Jousma, E., Wu, J., Rizvi, T. A., Brundage, M. E., Eaves, D., Widemann, B., Kim, M.-O., Dombi, E., et al. (2013). MEK inhibition exhibits efficacy in human and mouse neurofibromatosis tumors. *J. Clin. Invest.* **123**, 340–7.

Jopling, C., van Geemen, D. and den Hertog, J. (2007). Shp2 knockdown and Noonan/LEOPARD mutant Shp2-induced gastrulation defects. *PLoS Genet.* **3**, e225.

Kolanczyk, M., Kossler, N., Kühnisch, J., Lavitas, L., Stricker, S., Wilkening, U., Manjubala, I., Fratzl, P., Spörle, R., Herrmann, B. G., et al. (2007). Multiple roles for neurofibromin in skeletal development and growth. *Hum. Mol. Genet.* **16**, 874–86.

Kossler, N., Stricker, S., Rödelsperger, C., Robinson, P. N., Kim, J., Dietrich, C., Osswald, M., Kühnisch, J., Stevenson, D. A., Braun, T., et al. (2011). Neurofibromin (Nf1) is required for skeletal muscle development. *Hum. Mol. Genet.* **20**, 2697–709.

Krenz, M., Gulick, J., Osinska, H. E., Colbert, M. C., Molkentin, J. D. and Robbins, J. (2008). Role of ERK1/2 signaling in congenital valve malformations in Noonan syndrome. *Proc. Natl. Acad. Sci. U. S. A.* **105**, 18930–5.

Lakkis, M. M. and Epstein, J. A. (1998). Neurofibromin modulation of ras activity is required for normal endocardial-mesenchymal transformation in the developing heart. *Development* **125**, 4359–67.

Lakkis, M. M., Golden, J. A., O'Shea, K. S. and Epstein, J. A. (1999). Neurofibromin deficiency in mice causes exencephaly and is a modifier for Splotch neural tube defects. *Dev. Biol.* **212**, 80–92.

Largaespada, D. A., Brannan, C. I., Jenkins, N. A. and Copeland, N. G. (1996). Nf1 deficiency causes Ras-mediated granulocyte/macrophage colony stimulating factor hypersensitivity and chronic myeloid leukaemia. *Nat. Genet.* **12**, 137–43.

Lasater, E. A., Bessler, W. K., Mead, L. E., Horn, W. E., Clapp, D. W., Conway, S. J., Ingram, D. a and Li, F. (2008). Nf1+/- mice have increased neointima formation via hyperactivation of a Gleevec sensitive molecular pathway. *Hum. Mol. Genet.* **17**, 2336–44.

Lasater, E. A., Li, F., Bessler, W. K., Estes, M. L., Vemula, S., Hingtgen, C. M., Dinauer, M. C., Kapur, R., Conway, S. J. and Ingram, D. A. (2010). Genetic and cellular evidence of vascular inflammation in neurofibromin-deficient mice and humans. *J. Clin. Invest.* **120**, 859–870.

Le, L. Q., Shipman, T., Burns, D. K. and Parada, L. F. (2009). Cell of origin and microenvironment contribution for NF1-associated dermal neurofibromas. *Cell Stem Cell* **4**, 453–63.

Lee, D. Y., Yeh, T.-H., Emnett, R. J., White, C. R. and Gutmann, D. H. (2010a). Neurofibromatosis-1 regulates neuroglial progenitor proliferation and glial differentiation in a brain region-specific manner. *Genes Dev.* **24**, 2317–29.

Lee, J.-S., Padmanabhan, A., Shin, J., Zhu, S., Guo, F., Kanki, J. P., Epstein, J. a and Look, a T. (2010b). Oligodendrocyte progenitor cell numbers and migration are regulated by the zebrafish orthologs of the NF1 tumor suppressor gene. *Hum. Mol. Genet.* **19**, 4643–53.

Lee, D. Y., Gianino, S. M. and Gutmann, D. H. (2012). Innate neural stem cell heterogeneity determines the patterning of glioma formation in children. *Cancer Cell* **22**, 131–8.

Lee, Y.-S., Ehninger, D., Zhou, M., Oh, J.-Y., Kang, M., Kwak, C., Ryu, H.-H., Butz, D., Araki, T., Cai, Y., et al. (2014). Mechanism and treatment for learning and memory deficits in mouse models of Noonan syndrome. *Nat. Neurosci.* **17**, 1736–1743.

Li, W., Cui, Y., Kushner, S. A., Brown, R. A. M., Jentsch, J. D., Frankland, P. W., Cannon, T. D. and Silva, A. J. (2005). The HMG-CoA reductase inhibitor lovastatin reverses the learning and attention deficits in a mouse model of neurofibromatosis type 1. *Curr. Biol.* **15**, 1961–7.

Marin, T. M., Keith, K., Davies, B., Conner, D. A., Guha, P., Kalaitzidis, D., Wu, X., Lauriol, J., Wang, B., Bauer, M., et al. (2011). Rapamycin reverses hypertrophic cardiomyopathy in a mouse model of LEOPARD syndrome–associated PTPN11 mutation. *J. Clin. Invest.* **121**, 1026–1043.

Mayes, D. A., Rizvi, T. A., Cancelas, J. A., Kolasinski, N. T., Ciraolo, G. M., Stemmer-Rachamimov, A. O. and Ratner, N. (2011). Perinatal or adult Nf1 inactivation using tamoxifen-inducible PlpCre each cause neurofibroma formation. *Cancer Res.* **71**, 4675–85.

Miura, K., Wakayama, Y., Tanino, M., Orba, Y., Sawa, H., Hatakeyama, M., Tanaka, S., Sabe, H. and Mochizuki, N. (2013). Involvement of EphA2-mediated tyrosine phosphorylation of Shp2 in Shp2-regulated activation of extracellular signal-regulated kinase. *Oncogene* **32**, 5292–301.

Mohi, M. G., Williams, I. R., Dearolf, C. R., Chan, G., Kutok, J. L., Cohen, S., Morgan, K., Boulton, C., Shigematsu, H., Keilhack, H., et al. (2005). Prognostic, therapeutic, and mechanistic implications of a mouse model of leukemia evoked by Shp2 (PTPN11) mutations. *Cancer Cell* **7**, 179–91.

Nakamura, T., Colbert, M., Krenz, M., Molkentin, J. D., Hahn, H. S., Dorn, G. W. and Robbins, J. (2007). Mediating ERK1 / 2 signaling rescues congenital heart defects in a mouse model of Noonan syndrome. *J. Clin. Invest.* **117**, 2–11.

Nakamura, T., Gulick, J., Pratt, R. and Robbins, J. (2009). Noonan syndrome is associated with enhanced pERK activity, the repression of which can prevent craniofacial malformations. *Proc. Natl. Acad. Sci. U. S. A.* **106**, 15436–41.

Oishi, K., Gaengel, K., Krishnamoorthy, S., Kamiya, K., Kim, I.-K., Ying, H., Weber, U., Perkins, L. A., Tartaglia, M., Mlodzik, M., et al. (2006). Transgenic Drosophila models of Noonan syndrome causing PTPN11 gain-of-function mutations. *Hum. Mol. Genet.* **15**, 543–53.

Oishi, K., Zhang, H., Gault, W. J., Wang, C. J., Tan, C. C., Kim, I.-K., Ying, H., Rahman, T., Pica, N., Tartaglia, M., et al. (2009). Phosphatase-defective LEOPARD syndrome mutations in PTPN11 gene have gain-of-function effects during Drosophila development. *Hum. Mol. Genet.* **18**, 193–201.

Overman, J. P., Yi, J.-S., Bonetti, M., Soulsby, M., Preisinger, C., Stokes, M. P., Hui, L., Silva, J. C., Overvoorde, J., Giansanti, P., et al. (2014). PZR coordinates Noonan and LEOPARD syndrome signaling in zebrafish and mice. *Mol. Cell. Biol.* **34**, 2874–89.

Padmanabhan, A., Lee, J.-S., Ismat, F. A., Lu, M. M., Lawson, N. D., Kanki, J. P., Look, A. T. and Epstein, J. A. (2009). Cardiac and vascular functions of the zebrafish orthologues of the type I neurofibromatosis gene NFI. *Proc. Natl. Acad. Sci. U. S. A.* **106**, 22305–10.

Pagani, M. R., Oishi, K., Gelb, B. D. and Zhong, Y. (2009). The phosphatase SHP2 regulates the spacing effect for long-term memory induction. *Cell* **139**, 186–98.

Pong, W. W., Higer, S. B., Gianino, S. M., Emnett, R. J. and Gutmann, D. H. (2013). Reduced microglial CX3CR1 expression delays neurofibromatosis-1 glioma formation. *Ann. Neurol.* **73**, 303–8.

Razzaque, M. A., Komoike, Y., Nishizawa, T., Inai, K., Furutani, M., Higashinakagawa, T. and Matsuoka, R. (2012). Characterization of a novel KRAS mutation identified in Noonan syndrome. *Am. J. Med. Genet. A* **158A**, 524–32.

Reilly, K. M., Broman, K. W., Bronson, R. T., Tsang, S., Loisel, D. A., Christy, E. S., Sun, Z., Diehl, J., Munroe, D. J. and Tuskan, R. G. (2006). An Imprinted Locus Epistatically Influences Nstr1 and Nstr2 to Control Resistance to Nerve Sheath Tumors in a Neurofibromatosis Type 1 Mouse Model. *Cancer Res.* **66**, 62–68.

Rhodes, S. D., Wu, X., He, Y., Chen, S., Yang, H., Staser, K. W., Wang, J., Zhang, P., Jiang, C., Yokota, H., et al. (2013). Hyperactive transforming growth factor-β1 signaling potentiates skeletal defects in a neurofibromatosis type 1 mouse model. *J. Bone Miner. Res.* **28**, 2476–89.

Rosenbaum, T., Patrie, K. M. and Ratner, N. (1997). Neurofibromatosis Type 1 : Genetic and Cellular Mechanisms of Peripheral Nerve Tumor Formation. *Neurosci.* **3**, 412–420.

Runtuwene, V., van Eekelen, M., Overvoorde, J., Rehmann, H., Yntema, H. G., Nillesen, W. M., van Haeringen, A., van der Burgt, I., Burgering, B. and den Hertog, J. (2011). Noonan syndrome gain-of-function mutations in NRAS cause zebrafish gastrulation defects. *Dis. Model. Mech.* **4**, 393–9.

Santoriello, C., Deflorian, G., Pezzimenti, F., Kawakami, K., Lanfrancone, L., d'Adda di Fagagna, F. and Mione, M. (2009). Expression of H-RASV12 in a zebrafish model of Costello syndrome causes cellular senescence in adult proliferating cells. *Dis. Model. Mech.* **2**, 56–67.

Schindeler, A., Morse, A., Harry, L., Godfrey, C., Mikulec, K., McDonald, M., Gasser, J. A. and Little, D. G. (2008). Models of tibial fracture healing in normal and Nf1-deficient mice. *J. Orthop. Res.* **26**, 1053–60.

Schramm, C., Fine, D. M., Edwards, M. A., Reeb, A. N. and Krenz, M. (2012). The PTPN11 loss-of-function mutation Q510E-Shp2 causes hypertrophic cardiomyopathy by dysregulating mTOR signaling. *Am. J. Physiol. Heart Circ. Physiol.* **302**, 231–43.

Schuhmacher, A. J., Guerra, C., Sauzeau, V., Cañamero, M., Bustelo, X. R. and Barbacid, M. (2008). A mouse model for Costello syndrome reveals an Ang II – mediated hypertensive condition. *J. Clin. Invest.* **118**, 2169–2179.

Shilyansky, C., Karlsgodt, K. H., Cummings, D. M., Sidiropoulou, K., Hardt, M., James, A. S., Ehninger, D., Bearden, C. E., Poirazi, P., Jentsch, J. D., et al. (2010). Neurofibromin regulates corticostriatal inhibitory networks during working memory performance. *Proc. Natl. Acad. Sci. U. S. A.* **107**, 13141–6.

Shin, J., Padmanabhan, A., de Groh, E. D., Lee, J.-S., Haidar, S., Dahlberg, S., Guo, F., He, S., Wolman, M. A., Granato, M., et al. (2012). Zebrafish neurofibromatosis type 1 genes have redundant functions in tumorigenesis and embryonic development. *Dis. Model. Mech.* **5**, 881–94.

Silva, A. J., Frankland, P. W., Marowitz, Z., Friedman, E., Laszlo, G. S., Cioffi, D., Jacks, T., Bourtchuladze, R. and Lazlo, G. (1997). A mouse model for the learning and memory deficits associated with neurofibromatosis type I. *Nat. Genet.* **15**, 281–4.

Simmons, G. W., Pong, W. W., Emnett, R. J., White, C. R., Gianino, S. M., Rodriguez, F. J. and Gutmann, D. H. (2011). Neurofibromatosis-1 Heterozygosity Increases Microglia in a Spatially- and Temporally-Restricted Pattern Relevant to Mouse Optic Glioma Formation and Growth. *J Neuropathol Exp Neurol.* **70**, 51–62.

Sol-Church, K. and Gripp, K. W. (2009). The molecular basis of Costello syndrome. In *Noonan Syndrome and Related Disorders. Monogr Hum Genet.* (ed. Zenker, M.), pp. 94–103. Basel: Karger.

Solga, A. C., Gianino, S. M. and Gutmann, D. H. (2014). NG2-cells are not the cell of origin for murine neurofibromatosis-1 (Nf1) optic glioma. *Oncogene* **33**, 289–99.

Stewart, R. A., Sanda, T., Widlund, H. R., Zhu, S., Swanson, K. D., Hurley, A. D., Bentires-Alj, M., Fisher, D. E., Kontaridis, M. I., Look, A. T., et al. (2010). Phosphatase-dependent and -independent functions of Shp2 in neural crest cells underlie LEOPARD syndrome pathogenesis. *Dev. Cell* **18**, 750–62.

Sullivan, K., El-Hoss, J., Quinlan, K. G. R., Deo, N., Garton, F., Seto, J. T. C., Gdalevitch, M., Turner, N., Cooney, G. J., Kolanczyk, M., et al. (2014). NF1 is a critical regulator of muscle development and metabolism. *Hum. Mol. Genet.* **23**, 1250–9.

Sun, T., Gianino, S. M., Jackson, E., Piwnica-Worms, D., Gutmann, D. and Rubin, J. B. (2010). CXCL12 Alone Is Insufficient for Gliomagenesis in Nf1 Mutant Mice. **224**, 108–113.

Tajan, M., Batut, A., Cadoudal, T., Deleruyelle, S., Le Gonidec, S., Saint Laurent, C., Vomscheid, M., Wanecq, E., Tréguer, K., De Rocca Serra-Nédélec, A., et al. (2014). LEOPARD syndrome-associated SHP2 mutation confers leanness and protection from diet-induced obesity. *Proc. Natl. Acad. Sci. U. S. A.* **2**, 4494–4503.

Tartaglia, M. and Gelb, B. D. (2009). Molecular Genetics of Noonan Syndrome. In *Noonan Syndrome and Related Disorders - A Matter of Deregulated Ras Signaling. Monogr Hum Genet.* (ed. Zenker, M.), pp. 20–39. Basel: Karger.

The, I., Hannigan, G. E., Cowley, G. S., Reginald, S., Zhong, Y., Gusella, J. F., Hariharan, I. K. and Bernards, A. (1997). Rescue of a Drosophila NF1 mutant phenotype by protein kinase A. *Science* **276**, 791–4.

Tong, J., Hannan, F., Zhu, Y., Bernards, A. and Zhong, Y. (2002). Neurofibromin regulates G protein-stimulated adenylyl cyclase activity. *Nat. Neurosci.* **5**, 95–6.

Tong, J. J., Schriner, S. E., McCleary, D., Day, B. J. and Wallace, D. C. (2007). Life extension through neurofibromin mitochondrial regulation and antioxidant therapy for neurofibromatosis-1 in Drosophila melanogaster. *Nat. Genet.* **39**, 476–85.

Tuveson, D. a, Shaw, A. T., Willis, N. a, Silver, D. P., Jackson, E. L., Chang, S., Mercer, K. L., Grochow, R., Hock, H., Crowley, D., et al. (2004). Endogenous oncogenic K-ras(G12D) stimulates proliferation and widespread neoplastic and developmental defects. *Cancer Cell* **5**, 375–87.

Urosevic, J., Sauzeau, V., Soto-Montenegro, M. L., Reig, S., Desco, M., Wright, E. M. B., Cañamero, M., Mulero, F., Ortega, S., Bustelo, X. R., et al. (2011). Constitutive activation of B-Raf in the mouse germ line provides a model for human cardio-facio-cutaneous syndrome. *Proc. Natl. Acad. Sci. U. S. A.* **108**, 5015–20.

Van der Vaart, T., van Woerden, G. M., Elgersma, Y., de Zeeuw, C. I. and Schonewille, M. (2011). Motor deficits in neurofibromatosis type 1 mice: the role of the cerebellum. *Genes. Brain. Behav.* **10**, 404–9.

Viosca, J., Schuhmacher, A. J., Guerra, C. and Barco, A. (2009). Germline expression of H-Ras(G12V) causes neurological deficits associated to Costello syndrome. *Genes. Brain. Behav.* **8**, 60–71.

Vissers, L. E., Bonetti, M., Paardekooper Overman, J., Nillesen, W. M., Frints, S. G. M., de Ligt, J., Zampino, G., Justino, A., Machado, J. C., Schepens, M., et al. (2015). Heterozygous germline mutations in A2ML1 are associated with a disorder clinically related to Noonan syndrome. *Eur. J. Hum. Genet.* **23**, 317–324.

Vogel, K. S., Klesse, L. J., Velasco-Miguel, S., Meyers, K., Rushing, E. J. and Parada, L. F. (1999). Mouse tumor model for neurofibromatosis type 1. *Science* **286**, 2176–9.

Walker, J. A., Tchoudakova, A. V, McKenney, P. T., Brill, S., Wu, D., Cowley, G. S., Hariharan, I. K. and Bernards, A. (2006). Reduced growth of Drosophila neurofibromatosis 1 mutants reflects a non-cell-autonomous requirement for GTPase-Activating Protein activity in larval neurons. *Genes Dev.* **20**, 3311–23.

Walker, J. A., Gouzi, J. Y., Long, J. B., Huang, S., Maher, R. C., Xia, H., Khalil, K., Ray, A., Van Vactor, D., Bernards, R., et al. (2013). Genetic and functional studies implicate synaptic overgrowth and ring gland cAMP/PKA signaling defects in the Drosophila melanogaster neurofibromatosis-1 growth deficiency. *PLoS Genet.* **9**, e1003958.

Wang, S., Yu, W.-M., Zhang, W., McCrae, K. R., Neel, B. G. and Qu, C.-K. (2009). Noonan syndrome/leukemia-associated gain-of-function mutations in SHP-2 phosphatase (PTPN11) enhance cell migration and angiogenesis. *J. Biol. Chem.* **284**, 913–20.

Wang, W., Nyman, J. S., Ono, K., Stevenson, D. A., Yang, X. and Elefteriou, F. (2011). Mice lacking Nf1 in osteochondroprogenitor cells display skeletal dysplasia similar to patients with neurofibromatosis type I. *Hum. Mol. Genet.* **20**, 3910–24.

Wang, Y., Kim, E., Wang, X., Novitch, B. G., Yoshikawa, K., Chang, L.-S. and Zhu, Y. (2012). ERK inhibition rescues defects in fate specification of Nf1-deficient neural progenitors and brain abnormalities. *Cell* **150**, 816–30.

Warrington, N. M., Woerner, B. M., Daginakatte, G. C., Dasgupta, B., Perry, A., Gutmann, D. H. and Rubin, J. B. (2007). Spatiotemporal differences in CXCL12 expression and cyclic AMP underlie the unique pattern of optic glioma growth in neurofibromatosis type 1. *Cancer Res.* **67**, 8588–95.

Warrington, N. M., Gianino, S. M., Jackson, E., Goldhoff, P., Garbow, J. R., Piwnica-Worms, D., Gutmann, D. H. and Rubin, J. B. (2010). Cyclic AMP suppression is sufficient to induce gliomagenesis in a mouse model of neurofibromatosis-1. *Cancer Res.* **70**, 5717–27.

Williams, J. A., Su, H. S., Bernards, A., Field, J. and Sehgal, A. (2001). A circadian output in Drosophila mediated by neurofibromatosis-1 and Ras/MAPK. *Science* **293**, 2251–6.

Wolman, M. A., de Groh, E. D., McBride, S. M., Jongens, T. A., Granato, M. and Epstein, J. A. (2014). Modulation of cAMP and Ras Signaling Pathways Improves Distinct Behavioral Deficits in a Zebrafish Model of Neurofibromatosis Type 1. *Cell Rep.* **8**, 1–6.

Wozniak, D. F., Diggs-Andrews, K. A., Conyers, S., Yuede, C. M., Dearborn, J. T., Brown, J. A., Tokuda, K., Izumi, Y., Zorumski, C. F. and Gutmann, D. H. (2013). Motivational Disturbances and Effects of L-dopa Administration in Neurofibromatosis-1 Model Mice. *PLoS One* **8**, e66024.

Wu, J., Williams, J. P., Rizvi, T. A., Kordich, J. J., Witte, D., Meijer, D., Stemmer-rachamimov, A. O., Cancelas, J. A. and Ratner, N. (2008). Article Plexiform and Dermal Neurofibromas and Pigmentation Are Caused by Nf1 Loss in Desert Hedgehog-Expressing Cells. *Cancer Cell* **13**, 105–116.

Wu, X., Simpson, J., Hong, J. H., Kim, K., Thavarajah, N. K., Backx, P. H., Neel, B. G. and Araki, T. (2011a). MEK-ERK pathway modulation ameliorates disease phenotypes in a mouse model of Noonan syndrome associated with the Raf1 L613V mutation. *J. Clin. Invest.* **121**, 1009–1025.

Wu, X., Chen, S., He, Y., Rhodes, S. D., Mohammad, K. S., Li, X., Yang, X., Jiang, L., Nalepa, G., Snider, P., et al. (2011b). The haploinsufficient hematopoietic microenvironment is critical to the pathological fracture repair in murine models of neurofibromatosis type 1. *PLoS One* **6**, e24917.

Wu, X., Yin, J., Simpson, J., Kim, K.-H., Gu, S., Hong, J. H., Bayliss, P., Backx, P. H., Neel, B. G. and Araki, T. (2012). Increased BRAF heterodimerization is the common pathogenic mechanism for noonan syndrome-associated RAF1 mutants. *Mol. Cell. Biol.* **32**, 3872–90.

Xu, J., Ismat, F. A., Wang, T., Yang, J. and Epstein, J. A. (2007). NF1 regulates a Ras-dependent vascular smooth muscle proliferative injury response. *Circulation* **116**, 2148–56.

Xu, J., Ismat, F. A., Wang, T., Lu, M. M., Antonucci, N. and Epstein, J. A. (2009). Cardiomyocyte-specific loss of neurofibromin promotes cardiac hypertrophy and dysfunction. *Circ. Res.* **105**, 304–11.

Xu, D., Wang, S., Yu, W.-M., Chan, G., Araki, T., Bunting, K. D., Neel, B. G. and Qu, C.-K. (2010). A germline gain-of-function mutation in Ptpn11 (Shp-2) phosphatase induces myeloproliferative disease by aberrant activation of hematopoietic stem cells. *Blood* **116**, 3611–21.

Xu, D., Zheng, H., Yu, W.-M. and Qu, C.-K. (2013). Activating mutations in protein tyrosine phosphatase Ptpn11 (Shp2) enhance reactive oxygen species production that contributes to myeloproliferative disorder. *PLoS One* **8**, e63152.

Yang, F., Ingram, D. A., Chen, S., Zhu, Y., Yuan, J., Li, X., Yang, X., Knowles, S., Horn, W., Li, Y., et al. (2008). Nf1 -Dependent Tumors Require a Microenvironment Containing. *Cell* **135**, 437–448.

Yeh, T.-H., Lee, D. Y., Gianino, S. M. and Gutmann, D. H. (2009). Microarray analyses reveal regional astrocyte heterogeneity with implications for neurofibromatosis type 1 (NF1)-regulated glial proliferation. *Glia* **57**, 1239–49.

Yu, L., Daniels, J., Glaser, A. E. and Wolf, M. J. (2013). Raf-mediated cardiac hypertrophy in adult Drosophila. *Dis. Model. Mech.* **6**, 964–76.

Zhang, W., Rhodes, S. D., Zhao, L., He, Y., Zhang, Y., Shen, Y., Yang, D., Wu, X., Li, X., Yang, X., et al. (2011). Primary osteopathy of vertebrae in a neurofibromatosis type 1 murine model. *Bone* **48**, 1378–87.

Zhu, Y., Romero, M. I., Ghosh, P., Ye, Z., Charnay, P., Rushing, E. J., Marth, J. D. and Parada, L. F. (2001). Ablation of NF1 function in neurons induces abnormal development of cerebral cortex and reactive gliosis in the brain. *Genes Dev.* **15**, 859–76.

Zhu, Y., Ghosh, P., Charnay, P., Burns, D. K. and Parada, L. F. (2002). Neurofibromas in NF1: Schwann cell origin and role of tumor environment. *Science* **296**, 920–2.

Zhu, Y., Harada, T., Liu, L., Lush, M. E., Guignard, F., Harada, C., Burns, D. K., Bajenaru, M. L., Gutmann, D. H. and Parada, L. F. (2005). Inactivation of NF1 in CNS causes increased glial progenitor proliferation and optic glioma formation. *Development* **132**, 5577–5588.

Zhu, C., Smith, T., McNulty, J., Rayla, A. L., Lakshmanan, A., Siekmann, A. F., Buffardi, M., Meng, X., Shin, J., Padmanabhan, A., et al. (2011). Evaluation and application of modularly assembled zinc-finger nucleases in zebrafish. *Development* **138**, 4555–64.
